# Convergent evolution increases boron transport through SNPs and tandem duplications at *BOR1* and *BOR2* in *Arabidopsis thaliana*

**DOI:** 10.1073/pnas.2525676123

**Published:** 2026-03-23

**Authors:** Emmanuel Tergemina, Célia Neto, Md Mamunur Rashid, Herculano Dinis, David E. Salt, Angela M. Hancock

**Affiliations:** ^a^Department of Plant Developmental Biology, Max Planck Institute for Plant Breeding Research, Cologne 50829, Germany; ^b^Parque Natural do Fogo, Direção Nacional do Ambiente, Chã d’Areia, Praia, Santiago 115, Cabo Verde; ^c^Associação Projecto Vitó, Xaguate, Cidade de São Filipe, Fogo 8234, Cabo Verde; ^d^School of Biosciences, University of Nottingham, Sutton Bonington LE12 5RD, United Kingdom; ^e^Department of Botany and Plant Pathology, Purdue University, West Lafayette, IN 47907

**Keywords:** structural variation, tandem duplications, mineral transport, boron, GWAS

## Abstract

Boron (B) is a crucial micronutrient, particularly in volcanic soils where its deficiency hampers agriculture. Here, we investigate the genetic basis of leaf B accumulation in natural populations of *Arabidopsis thaliana* that colonized volcanic islands in Cape Verde. Using a combination of genome-wide association studies (GWAS) and mapping in a recombinant intercross population, we identified a case of convergent phenotypic evolution in which multiple variants in the two principal B transporter genes, *BOR1* and *BOR2*, increase leaf B accumulation in parallel. These include multiple tandem duplications at *BOR1* that arose independently in different populations. Overall, this study reveals a remarkable case of convergent evolution occurring within a relatively short time scale, where different types of de novo mutations at B efflux transporter genes achieve similar phenotypic outcomes. Further, our findings show that integrating recombinant populations with GWAS in natural populations can improve power to overcome allelic heterogeneity.

## Significance

Boron is an essential nutrient for plant growth, but many soils lack sufficient amounts, limiting crop production worldwide. To understand how plants adapt to boron-deficient environments, we studied wild mustard plants that naturally colonized volcanic islands, where boron is typically scarce. We identified a case of convergent evolution, wherein different plant populations independently evolved to increase boron concentrations in their leaves through distinct genetic changes in genes controlling boron transport. This finding reveals a key biological mechanism that may help plants survive in boron-poor soils, providing valuable insights for developing more resilient crops that can thrive in challenging agricultural environments.

Boron (B) is an essential micronutrient in plants, playing a crucial role in cellular and physiological processes. In particular, this element is vital for forming and maintaining cell walls because it covalently links pectic polysaccharides rhamnogalacturonan-II (RG-II) chains, forming a network that is necessary for cell wall adhesion and structure (1–4). The distance between B deficiency and excess in plants is the narrowest of all nutrients (5). While B deficiency induces cell wall abnormalities, growth defects, and sometimes sterility, B excess causes DNA damage and tissue necrosis (6–8).

Boric acid is the main form of B available to plants in soil solution. As a small and uncharged molecule, boric acid is able to permeate lipid bilayers, which allows it to diffuse passively through the plasma membrane when soil B levels are adequate (9). However, under low soil B conditions, plants rely on two additional mechanisms to transport B across the plasma membrane: facilitated diffusion of boric acid through channels of the nodulin 26-like intrinsic proteins (NIPs) and active transport of borate (formed in the cytoplasm) through efflux transporters of the BOR family (10). In *Arabidopsis thaliana*, the primary boric acid channel and borate transporter contributing to B homeostasis are NIP5;1 and BOR1, respectively (11, 12). NIP5;1 and BOR1 localize asymmetrically in the plasma membrane of root cells, with a soil-side orientation of NIP5;1 to facilitate B uptake into root cells and a stele-side orientation of BOR1 for B xylem loading (13). This polar localization enables transcellular transport of B to the xylem from the soil solution, allowing B to reach the upper part of plants through the transpiration stream, particularly under B limitation. However, when B is present in sufficient amounts or in excess, the activities of BOR1 and NIP5;1 are downregulated to prevent toxicity. NIP5;1 downregulation depends on ribosome stalling (14, 15), while BOR1 downregulation relies on protein degradation and translational repression (16–18). Therefore, B transporter activity is tightly regulated at multiple levels to maintain B homeostasis.

B-deficient soils severely restrict agriculture worldwide (19). The occurrence of B deficiency in soil is due to several factors, including the composition of the parent material, pH, structure, weathering, and leaching processes. In particular, crops growing in volcanic soils are often B-deficient, primarily due to the high B-adsorption capacity of volcanic ash-derived soils (19–25). Here, we examine the evolution of leaf B accumulation in *A. thaliana* from two drought-prone volcanic islands in Cape Verde. Using a combination of genome-wide association studie (GWAS) in the natural population and mapping in a constructed recombinant population, we identify multiple variants in B transporter genes that increase B accumulation in leaf tissues. Then, we reconstruct their spread across the islands.

## Results

**Fig. 1.**
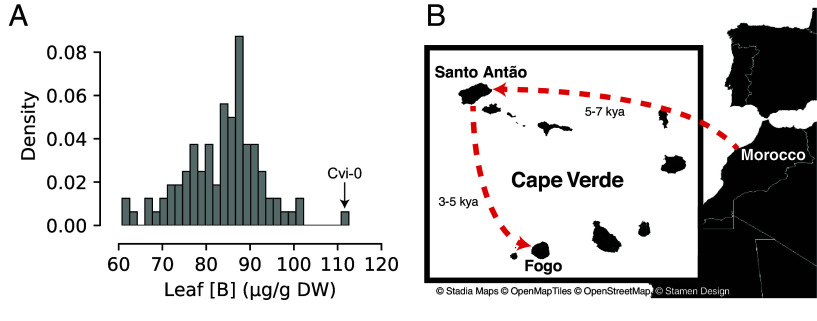
Cvi-0 is an outlier in the worldwide distribution of Leaf B in *Arabidopsis thaliana.* (*A*) Leaf B accumulation in μg/g of dry weight (DW) in a worldwide panel of *A. thaliana* accessions. (*B*) Historical reconstruction indicates that *A. thaliana* colonized Cape Verde in Santo Antão first (approximately 5 to 7 thousand years ago) from a population originating in North Africa, best represented by the current Moroccan population, and Fogo was colonized from Santo Antão (approximately 3 to 5 thousand years ago).

Cvi-0, an *A. thaliana* accession collected in the Cape Verde Islands (CVI), is among the accessions with the highest levels of leaf B accumulation compared to a worldwide diversity panel of *A. thaliana* (26) under B-sufficient conditions, indicating that B transport to leaves is particularly efficient in this accession (Fig. 1*A*). *A. thaliana* is present in Cape Verde in two islands, Santo Antão and Fogo. Previous historical reconstructions indicated that *A. thaliana* colonized the two islands approximately five thousand years ago (kya) from a population originating in North Africa, best represented by the current Moroccan population (Fig. 1*B*) (27). Colonization of each island is associated with a severe bottleneck that erased most of the standing genetic variation. As a result, the two island populations are phylogenetically distinct, and the genetic variation that contributes to trait variation in each Cape Verde island arose de novo after the colonization of the islands.

### GWAS Identifies Multiple Loci That Increase Leaf B Accumulation in Cape Verde.

We characterized the genetic architecture of leaf B accumulation within each CVI population in a standard potting mix (Dataset S1). First, we estimated broad-sense heritability (H^2^) based on repeatability across replicates and narrow-sense heritability based on the phenotypic variance explained by available genotypes (PVE) (28). Then, to identify genetic variants that contribute to variation in B accumulation, we conducted GWAS using a linear mixed model (LMM) approach that controls for population structure through the inclusion of a relatedness matrix (29).

**Fig. 2.**
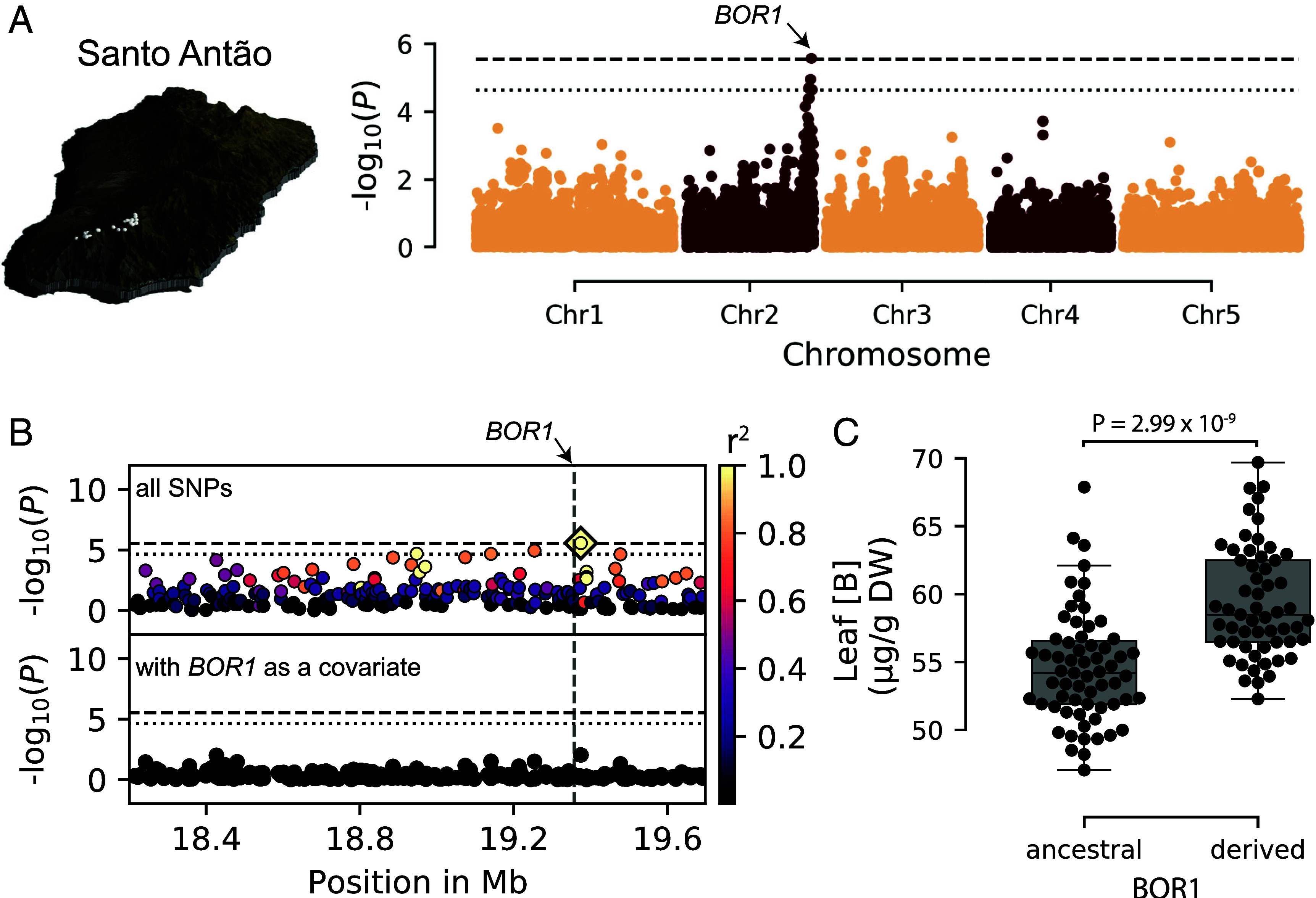
A variant at the *BOR1* region increases leaf B in Santo Antão. (*A*) genome-wide association studie (GWAS) of leaf B in Santo Antão. The chromosomes are color-coded. The dashed horizontal line indicates the 5% Bonferroni-adjusted genome-wide significance threshold while the dotted horizontal line corresponds to the 10% false discovery rate (FDR) threshold. (*B*) Magnification of the chromosome 2 peak. Unconditioned GWAS (*Upper* panel) and GWAS conditioned on the *BOR1* region (chr2:19374681) (*Lower* panel). Colors represent the extent of LD with the most significant marker (chr2:19374681), indicated with a diamond. The dashed horizontal lines correspond to the 5% Bonferroni-adjusted genome-wide significance threshold and the dotted horizontal lines indicate the 10% FDR threshold. The vertical dashed line indicates *BOR1*. (*C*) Leaf B concentration in samples with the two *BOR1* haplotypes in µg/g of dry weight. Each dot represents an accession. *P* = *P* value for Mann–Whitney–Wilcoxon (MWW) test.

In Santo Antão, using 123 accessions carrying 17,436 variants after filtering, we found that leaf B accumulation was moderately heritable, based on the proportion of the variation explained by genetic factors (H^2^ = 38.9%, PVE median = 70.4%, 95% CI = 53.2 to 97.5). We identified a significant peak associated with variation in leaf B concentration at the end of chromosome 2 coinciding with *BOR1* (AT2G47160) (Fig. 2*A*). *BOR1* is an excellent candidate because it encodes the major B efflux transporter loading xylem for root-to-shoot translocation in B-deficient conditions (11). The most significant SNP is located approximately 17 kb downstream of *BOR1*. We manually examined the region but did not identify a clear functional coding variant or evidence of structural variation in *BOR1* (Dataset S2), suggesting that nearby regulatory variation may be responsible for the signal. When we conditioned on the most significant SNP, no signal remained, suggesting that a single *BOR1* haplotype can explain the signal in the region (Fig. 2*B* and *SI Appendix*, Fig. S1). B accumulation was higher in Santo Antão accessions carrying the derived *BOR1* haplotype [Mann–Whitney–Wilcoxon (MWW) test, *P* = 2.99 × 10^−9^] (Fig. 2*C*). Using the LMM, we estimated that the derived *BOR1* haplotype was associated with an increase in leaf B by approximately 6.62 µg/g of dry weight (DW). Overall, these results suggest that a mutation in the *BOR1* region increases leaf B accumulation in the derived state in *A. thaliana* in Santo Antão.

**Fig. 3.**
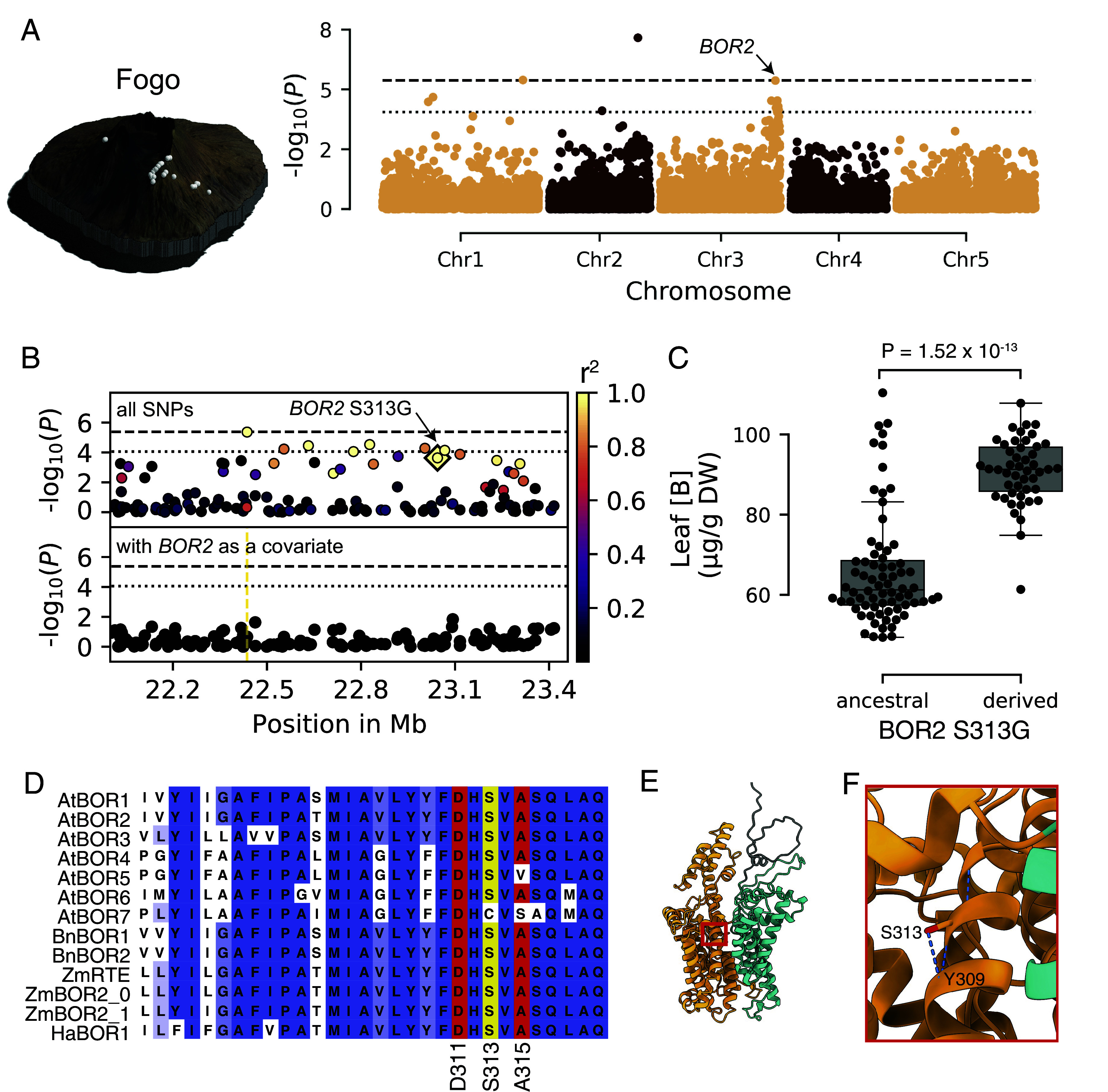
A nonsynonymous variant at *BOR2* associated with an increase in leaf B in Fogo. (*A*) GWAS of leaf B in Fogo. The chromosomes are color-coded. The dashed horizontal line corresponds to the 5% Bonferroni-adjusted genome-wide significance threshold. The dotted horizontal line indicates the 10% FDR threshold. (*B*) Magnification of the chromosome 3 peak. Unconditioned GWAS (*Upper* panel) and GWAS conditioned on the most significant marker (chr3:22436584) (*Lower* panel). Colors indicate the extent of LD with chr3:22436584. The dashed horizontal lines correspond to the 5% Bonferroni-adjusted genome-wide significance threshold. The dotted horizontal lines indicate the 10% FDR threshold. The diamond shows the *BOR2 S313G* variant. (*C*) Leaf B concentration of plants with the two *BOR2 S313G* alleles in µg/g of DW. Each dot represents an accession. *P* = *P* value for MWW test. (*D*) Protein sequence alignment at the 8th transmembrane domain of the BOR family in angiosperms (At: *Arabidopsis thaliana*, Bn: *Brassica napus*, Zm: *Zea mays*, Ha: *Helianthus annuus*). *BOR2 S313G* is highlighted in yellow. The mutations known to affect the core domain of BOR1 are highlighted in red. (*E*) Structural prediction of the BOR2 protein. The core domain is indicated in orange, and the gate domain is indicated in cyan. (*F*) magnification showing the hydrogen bond between the side chain of S313 and the main chain of Y309.

In Fogo, using 126 accessions harboring 11,939 variants after filtering, we found leaf B accumulation was highly heritable, based on the proportion of the variation explained by genetic factors (H^2^ = 75%, PVE median = 84.4%, 95% CI = 75.9 to 91). An LMM revealed two isolated Bonferroni significant markers, one at the end of chromosome 1, and one at the end of chromosome 2 located approximately 200 kb upstream of *BOR1* as well as a peak on chromosome 3 that coincided with *BOR2* (AT3G62270) (Fig. 3*A*). The most strongly associated marker in this region was in perfect LD (*r*^2^ = 1) with a nonsynonymous variant (S313G) in *BOR2* that is private to Fogo (Dataset S3). *BOR2* is a strong candidate because it is a close paralog to *BOR1,* and it has been shown to contribute to B transport to leaf tissue (30). While the markers on chromosomes 1 and 2 remained Bonferroni significant after conditioning for the most strongly associated marker at the *BOR2* region, no signal remained at *BOR2* itself, which indicated that a single haplotype can explain the signal in that region (Fig. 3*B* and *SI Appendix*, Fig. S2). Fogo accessions carrying the derived *BOR2* haplotype accumulated more B in leaf tissue (MWW test, *P* = 1.52 × 10^−13^) (Fig. 3*C*). Using the LMM, we inferred that the derived *BOR2* haplotype increased leaf B by 22.72 µg/g of DW. To assess the potential impact of this *BOR2 313G* mutation, we examined the AlphaFold2 predicted structure of BOR2. Because BOR1 and BOR2 protein sequences share 90% homology (30), the predicted structure of BOR2 closely matches BOR1 (*SI Appendix*, Fig. S3*A*) (31). The BOR1 crystal structure has been resolved and indicates that the protein adopts a homodimeric conformation (32). Each monomer contains two gate domains for dimerization and two core domains containing the putative substrate-binding site. *BOR2 313G* is located in the core domain within the 8th transmembrane domain at a highly conserved position in *BOR* orthologs (Fig. 3*D*). Based on the predicted structure of BOR2, *BOR2 313G* breaks a buried hydrogen bond between the side chain of S313 and the main chain of Y309, suggesting that the mutation induces a conformational change within its core domain (Fig. 3 *E* and *F*). Previous genetic screens of BOR1 indicate that mutations in this transmembrane domain can prevent degradation of the protein by inhibiting its ubiquitination and subsequent transport to the vacuole (18) (Fig. 3*D*). Similar to BOR1, BOR2 accumulates in root cells at the plasma membrane with stele side polarity in low B conditions and is directed to the vacuole for degradation in higher B conditions (17, 30). Therefore, it is plausible that *BOR2 313G* impedes the degradation of BOR2. Overall, our results reveal multiple genomic regions associated with leaf B variation in Fogo, with a clear signal at *BOR2*. Furthermore, the *BOR2 313G* variant is a strong candidate to explain the signal on chromosome 3.

### Mapping in a CVI Multiparent Intercross Population Improves Power to Identify Causal Variants.

**Fig. 4.**
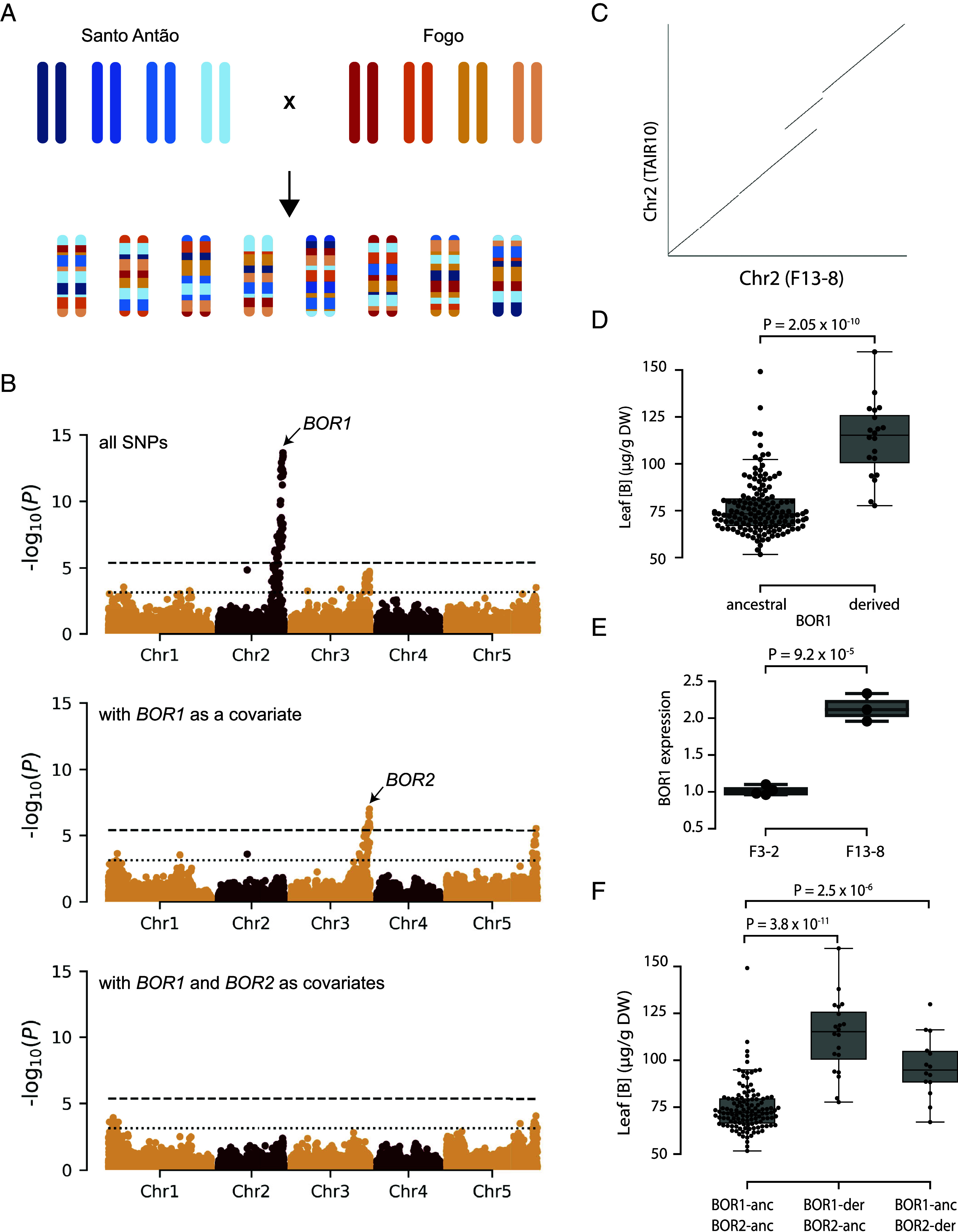
Mapping leaf B accumulation in an intercross population reveals a tandem duplication at *BOR1* in Fogo. (*A*) Representation of the CVI intercross population. (*B*) Leaf B accumulation mapping in the CVI intercross population. Unconditioned GWAS (*Upper* panel), GWAS conditioned on the *BOR1* region (chr2:19379353) (*Middle* panel), and GWAS conditioned on the *BOR1* (chr2:19379353) and the *BOR2* (chr3:23254270) regions (*Lower* panel). The chromosomes are color-coded. The dashed horizontal lines correspond to the 5% Bonferroni-adjusted genome-wide significance threshold, and the dotted horizontal lines indicate the 10% FDR threshold. (*C*) Dot-plots at *BOR1* in F13-8 show a tandem duplication. The x and y axes correspond to the position at chromosome 2 for de novo assembled genome of F13-8 and the TAIR10 reference, respectively. (*D*) Leaf B concentration in plants with the two *BOR1* CN alleles in µg/g of DW. Each dot represents a CVI intercross individual. *P* = *P* value for MWW test. (*E*) F13-8 (two *BOR1* copies) shows higher *BOR1* mRNA levels in the roots compared to F3-2 (one *BOR1* copy). *BOR1* mRNA levels were quantified with a probe-based digital PCR assay. *P* = *P* value for *t* test. (*F*) Variation in leaf B concentration in the CVI intercross population with allelic combination between *BOR1* and *BOR2*. anc = ancestral, der = derived. We did not observe any doubled-haploid individuals carrying *BOR1*-der and *BOR2*-der. Results are shown in µg/g of DW. Each dot represents an individual. *P* = *P* value for MWW test using individuals carrying the ancestral alleles at *BOR1* and *BOR2* as control.

The power of detection from GWAS is known to be limited by population structure and allelic heterogeneity (33). However, constructed recombinant populations have the potential to capture much of the common variation in a natural population while overcoming population structure (34). To identify other variants that impact leaf B accumulation in CVI, we complemented our population-based mapping approach with mapping in an eight-parent intercross population we produced from crosses between Fogo and Santo Antão plants (35) (Fig. 4*A* and Dataset S4). Using 167 CVI-intercross individuals carrying 11,682 variants after filtering, we found that a high proportion of the variation in leaf B in the intercross population could be explained by genetic factors (H^2^ = 63.6%, PVE median = 68.3%, 95% CI = 51.3 to 82.9). An LMM revealed a single Bonferroni significant peak on chromosome 2 coinciding with *BOR1* (Fig. 4*B*). No signal remained in the chromosome 2 region once we conditioned for the most significant marker, implying that a single *BOR1* haplotype was sufficient to explain the signal in the region. In addition, we found that among the eight parents of the intercross populations, this derived *BOR1* haplotype was found only in the Fogo individual F13-8. Since no SNP or INDEL variant in the *BOR1* coding region appeared likely to have a functional effect on BOR1, we examined structural variation in this genomic region using short-read sequencing data from F13-8. We identified an approximately 38 kb tandem duplication (TD) containing *BOR1*, which we confirmed using a whole-genome de novo assembly we generated from Nanopore sequencing data for F13-8 (Fig. 4*C*). Leveraging a recombinant map for the CVI intercross population (35), we found that the most significant SNP (chr2:19379353) is in perfect LD (*r*^2^ = 1) with the TD at *BOR1* in the intercross population (Dataset S5). CVI recombinant individuals carrying the TD at *BOR1* accumulated more B in their leaves (MWW test, *P* = 2.05 × 10^−10^; Fig. 4*D*). The effect size of the Fogo TD was approximately five times greater than that of the *BOR1* haplotype that we identified in the Santo Antão population (CVI intercross: beta = 34.82 µg/g of DW, Santo Antão: beta = 6.62 µg/g of DW). Together, our results supported the presence of at least two distinct *BOR1* haplotypes segregating in Cape Verde.

An increase in *BOR1* copy number (CN) represents an excellent functional candidate because lines overexpressing *BOR1* are known to accumulate more B in the shoots (36, 37). Therefore, we examined whether the gain in *BOR1* CN was linked with increased *BOR1* expression in root tissue. We found an approximately twofold higher *BOR1* mRNA level in the F13-8 accession compared to a Fogo accession with a single *BOR1* copy (F3-2) (Student’s *t* test, *P* = 9.2 × 10^−5^) (Fig. 4*E*). Our results show that the TD at *BOR1* carried by the accession F13-8 can explain the chromosome 2 peak we observed in the CVI intercross population.

In addition to the *BOR1* peak, mapping for leaf B variation in the CVI intercross population revealed a peak at the distal end of chromosome 3. While this peak was not Bonferroni significant, it met a less stringent FDR threshold of 10% and coincided with *BOR2*. After conditioning for the *BOR1* region, the peak at the *BOR2* region became Bonferroni significant, along with another signal at the end of chromosome 5 (Dataset S6). We examined the genomic region corresponding to the peak at the distal end of chromosome 5 but found no genes previously shown to be involved in B homeostasis. Potential candidates in the region include *RECEPTOR HOMOLOGY REGION TRANSMEMBRANE DOMAIN RING H2 MOTIF PROTEIN 1* (*RMR1*, AT5G66160), a gene encoding a ring H2 motif protein involved in protein trafficking to the protein storage vacuole (38).

The *BOR2* peak in the intercross population is defined by a haplotype containing the *BOR2 313G* variant that we identified in the Fogo natural population (Fig. 4*B*). This variant is present in the intercross population due to the parental line F3-2. Similar to what we found in the Fogo natural population, the signal at *BOR2* disappeared when we added the most significant marker in the region as a covariate in the LMM conditioned for *BOR1* TD, indicating that a single *BOR2* haplotype accounted for the signal at *BOR2* (Fig. 4*B*). Consistent with the GWAS results obtained in the Fogo population, CVI recombinant individuals carrying the *BOR2 313G* allele accumulated more B in the leaves (MWW test, *P* = 2.5 × 10^−6^; Fig. 4*F*) with an estimated effect size comparable to that in the Fogo population (CVI intercross: beta = 22.61 µg/g of DW after conditioning for *BOR1* TD, Fogo: beta = 22.72 µg/g of DW). Overall, our findings in the CVI intercross population reveal a *BOR1* TD haplotype in Fogo that increases leaf B and a new genomic region at the distal end of chromosome 5 involved in B homeostasis. Additionally, these results reinforce the role of *BOR2 S313G* in leaf B variation.

### Multiple *BOR1* Tandem Duplications Arose Independently in Cape Verde.

**Fig. 5.**
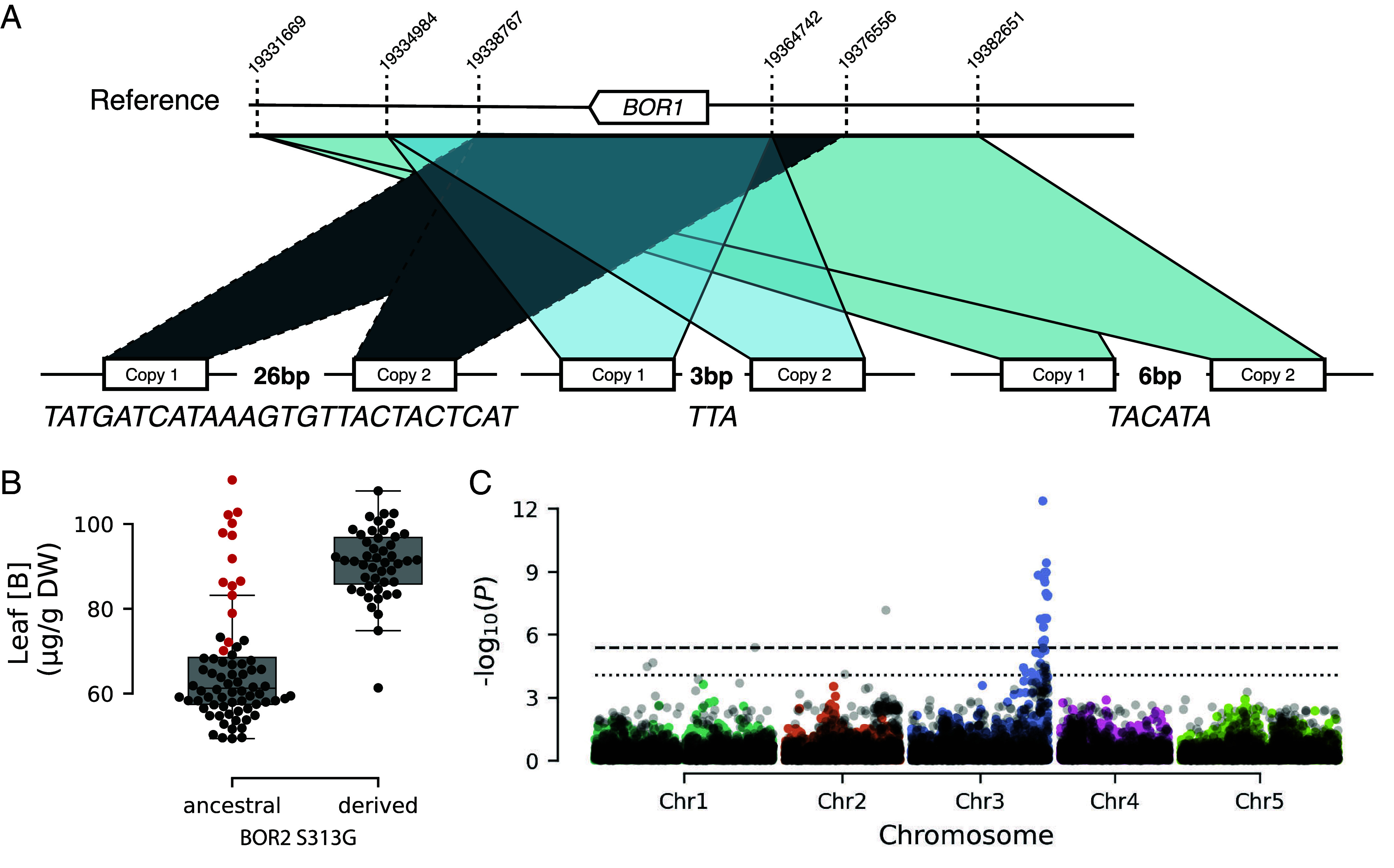
Three independent tandem duplication events at *BOR1* increase B in the leaves in Fogo. (*A*) Schematic representation of three TD events at *BOR1*. The numbers above the dashed vertical lines indicate the position on chromosome 2 of the TAIR10 reference genome. (*B*) Difference in leaf B concentration between the two *BOR2 S313G* alleles in the Fogo population. Fogo plants carrying an extra *BOR1* copy are indicated in red. Results are shown in µg/g of DW. Each dot represents an accession. (*C*) GWAS of leaf B variation in the Fogo population with *BOR1* CN as a covariate. The original GWAS is plotted in black. GWAS using *BOR1* CN as a covariate is plotted with color-coded chromosomes. The dashed horizontal line corresponds to the 5% Bonferroni-adjusted genome-wide significance threshold, and the dotted horizontal line indicates the 10% FDR threshold.

We further examined the *BOR1* region across the Fogo natural population and found evidence for multiple tandem duplications, each with distinct nonoverlapping breakpoints. The TD breakpoint junctions reveal microhomology of three and six bp (*TTA* and *TACATA,* referred to as *chr2:19331669* and *chr2:19334984* in subsequent text) and a novel sequence of 26 bp (*TATGATCATAAAGTGTTACTACTCAT* referred to as *chr2:19338767)* (Fig. 5*A* and *SI Appendix*, Figs. S4–S8 and Dataset S7). The presence of microhomology and a novel sequence at the TD breakpoint junctions suggests that the TDs may have arisen from error-prone repair processes following replication stress or DNA double-strand breaks, such as microhomology-mediated break-induced replication or end-joining mechanisms (39).

Using short-read sequencing data, we found that the Fogo plants carry a maximum of two *BOR1* copies, which we confirmed in a subset of lines using quantitative digital PCR (dPCR) (*SI Appendix*, Fig. S9 and Dataset S8). Consistent with our results from the CVI intercross population, Fogo plants carrying an extra *BOR1* copy accumulated more B in the leaves (MWW test, *P* = 6 × 10^−9^) (*SI Appendix*, Fig. S10). These TDs also helped explain why the outliers from Fig. 3*C* carrying the *BOR2 S313G* ancestral allele had high B levels (Fig. 5*B*).

The high number of independent TD events at *BOR1* is remarkable, especially given the relatively recent colonization of Fogo (~3 to 5 kya) and the low SNP diversity (*θ*_w(Fogo)_ = 8.93 × 10^−5^). To determine whether TDs at *BOR1* can be found in other *A. thaliana* populations, we gathered published *A. thaliana* sequence data (27, 40–43) and looked for evidence of TDs in the *BOR1* region across all worldwide data, including CVI. We found no evidence of TDs that include the entire *BOR1* coding sequence outside of CVI. However, we identified an additional TD within Santo Antão. This TD was at low frequency on the island, present in only five accessions from two stands in Santo Antão. The breakpoints are different from the TDs we observed in Fogo, indicating that this TD arose independently (*SI Appendix*, Fig. S11). These results indicate that TDs at *BOR1* are common in CVI but also unique to these islands.

Next, we investigated how *BOR1* CN affects our GWAS in the Fogo population. Using *BOR1* CN as a covariate, the signal at *BOR2* improved (Fig. 5*C*), and the inferred effect of *BOR2 313G* on leaf B increased (beta = 26.85 g/g of DW with *BOR1* CN as a covariate, beta = 22.72 µg/g of DW without *BOR1* CN as a covariate). Together, these results show that although *BOR1* TDs were weakly detectable using GWAS for leaf B in Fogo, power was increased to detect variation at both *BOR1* and *BOR2*. This result highlights the benefits of complementing natural populations with recombinant populations for trait mapping.

We next examined the evolutionary history of the *BOR1* and *BOR2* variants in Santo Antão and Fogo. In Santo Antão, the *BOR1 chr2:19374681* haplotype identified in GWAS segregates at intermediate frequency (46%) on the island (*SI Appendix*, Fig. S12*A*) while the *BOR1* TD haplotype segregates at 2.6% on the island and is private to the Pico subpopulation (*SI Appendix*, Fig. S13*A*). We conducted coalescent modeling (44) to reconstruct the history of the *BOR1* region in Santo Antão (Dataset S9). We found that the mutations at *BOR1* arose after the expansion into the Espongeiro and Pico regions [approximately 3 kya (27)], between 1.4 kya [95% CI (CI): 661 y to 2.2 kya], based on the estimated time to the most recent common ancestor (tMRCA), and 2.47 kya (95% CI: 1.9 to 2.97 kya) based on the allelic divergence estimate for the *BOR1 chr2:19374681* haplotype, and between 90 y (95% CI: 36 to 177 y) and 207 y (95% CI: 102 to 378 y) for the *BOR1* TD haplotype (*SI Appendix*, Figs. S12*B* and 13*B*).

In Fogo, the three *BOR1* TD haplotypes have a combined frequency of 11.6% in the island. Each *BOR1* TD haplotype is private to a different Fogo subpopulation, leading to a clear geographical demarcation of the TDs across the island (*SI Appendix*, Fig. S14*A*). In accordance with their moderate frequencies, coalescent reconstruction of the *BOR1* region indicates that the TDs arose recently, well after the split between subpopulations, between 185 y (95% CI: 60 to 417 y) and 2.72 kya (95% CI: 2.18 to 3.19 kya) for the *chr2:19338767* TD haplotype, between 106 y (95% CI: 15 to 365 y) and 1.90 kya (95% CI: 1.58.8 to 2.22 kya) for the *chr2:19331669* TD haplotype, and between 145 y (95% CI: 21 to 528 y) and 1.76 kya (95% CI: 1.46 to 2.07 kya) for the *chr2:19334984* TD haplotype (*SI Appendix*, Fig. S14*B*). The geographical distributions and age estimates of the TD haplotypes are consistent with the lack of evidence for recombination between haplotypes.

The *BOR2 313G* variant segregates at 42% in Fogo and is present in Monte Velha and Inferno regions but is absent from the Lava region (*SI Appendix*, Fig. S15*A*). Coalescent reconstruction at the *BOR2* region indicated that the *BOR2 313G* variant arose approximately between 1.38 kya (95% CI: 654 y to 2.37 kya) and 2.76 kya (95% CI: 2.2 to 3.25 kya), which coincide with the period when structure was beginning to develop on the island [approximately 1.6 to 2.0 kya (45)] (*SI Appendix*, Fig. S15*B*). Overall, the occurrence of multiple independent mutations at *BOR1* and *BOR2* cause phenotypic convergence toward increased B transport to leaf tissue during the spread across the islands.

## Discussion

By mapping variation in B accumulation in CVI natural and recombinant populations, we identified multiple haplotypes associated with increased leaf B. We identified five *BOR1* haplotypes, including four TDs associated with increased expression of the gene, and one *BOR2* haplotype. Overall, this is consistent with a model in which multiple variants that increase B transport arose and increased in frequency in parallel across Cape Verde Island populations.

Our results have implications for mapping by GWAS in natural populations. The power of detection in GWAS is strongly affected by allelic heterogeneity and population structure, which may be common in nature (33, 46, 47). In our study, allelic heterogeneity at *BOR1* both impeded the detection of that locus and reduced the power to detect variation at *BOR2* through GWAS. In structured natural populations, different subpopulations may adapt through new variants that arise at different sites within the same gene and different genes across the genome, resulting in what has been termed a soft selective sweep (48). Subsequent gene flow may help homogenize the populations, but the resulting pattern of allelic and genetic heterogeneity may reduce power in GWAS. With this study, we show that joint analysis of natural and recombinant populations helps to overcome these problems. Given that the CVI populations are young and have a simple history relative to older continental populations, we expect the case in continental populations to be even more challenging. Overall, our results imply that allelic heterogeneity may be common in natural populations and could reduce the effectiveness of GWAS.

**Fig. 6.**
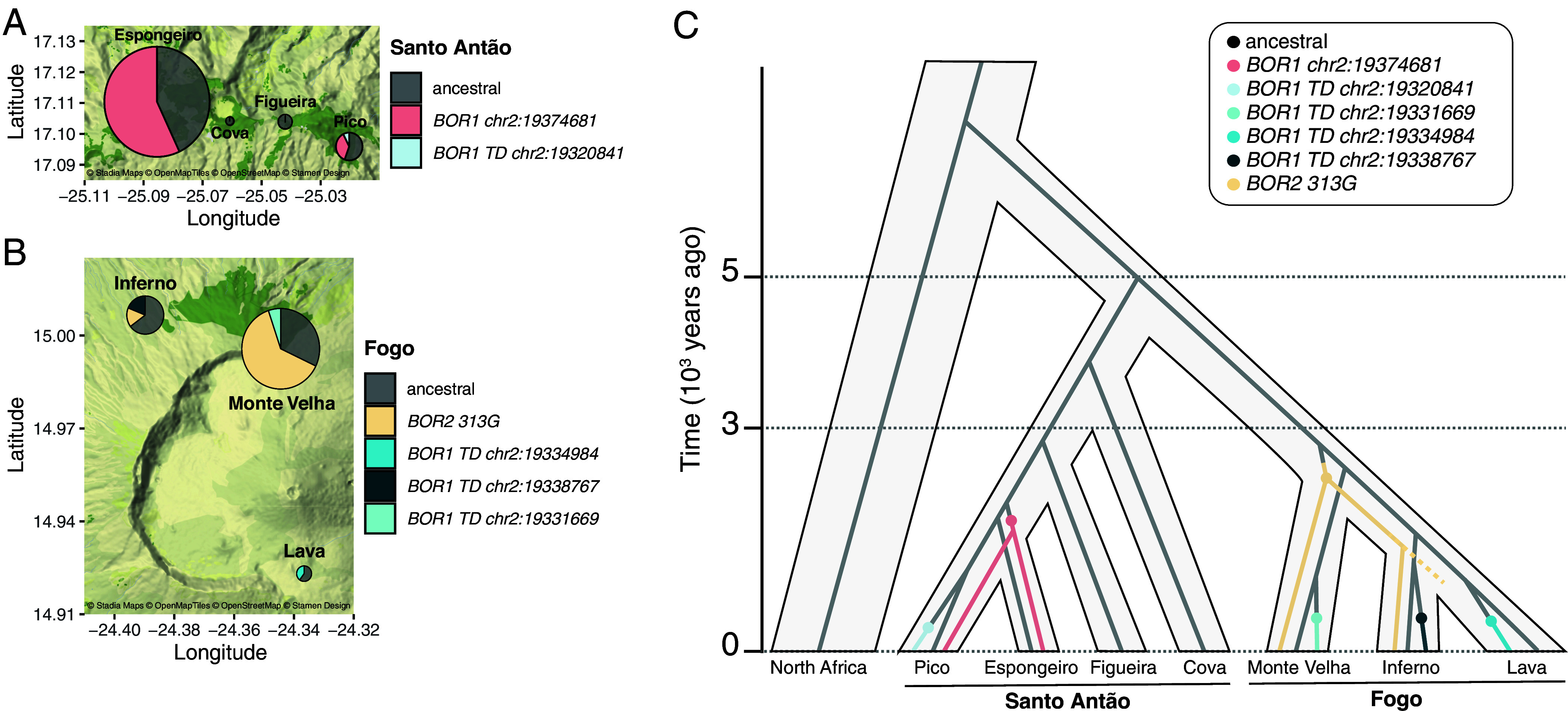
History of *BOR1* and *BOR2* in Cape Verde. Geographic distribution of the *BOR1* and *BOR2* haplotypes identified in Santo Antão (*A*) and Fogo (*B*). (*C*) Evolutionary model of *BOR1* and *BOR2* in Cape Verde.

Taken together, our findings indicate that B accumulation increased due to convergent evolution from multiple alleles that arose independently in the Cape Verde Island populations (Fig. 6). The selection pressures that drove these convergent changes may be due to a combination of climatic and edaphic factors. The growing season in Cape Verde is short due to seasonal drought and begins with the rainy season, characterized by heavy rainfall that could leach B from the soils (19). In addition, the soils in Cape Verde are of volcanic origin, which typically have a high capacity for B adsorption (19–25). Fogo island, in particular, has a high rate of volcanic activity, with the most recent eruption in 2014, and has a drier climate than Santo Antão. The combination of more recent volcanic activity and higher prevalence of drought on Fogo relative to Santo Antão suggests the selective pressure to increase B transport to leaf tissue may have been stronger on that island. These factors offer a potential explanation for the higher number of observed de novo mutations and larger effect sizes in Fogo. Stronger selective pressure in Fogo may have created a situation where de novo mutations that arose were more likely to escape drift or purifying selection.

Increases in gene copy number represent an important mode of rapid trait evolution. In Cape Verde, four independent TDs arose at *BOR1* in different subpopulations and rose in frequency in parallel (Figs. 5 and 6). In contrast, we could detect no evidence of TDs containing *BOR1* in 1,646 individuals collected from other parts of the world. This suggests that TDs that arose in this genomic region in other populations were removed by purifying selection, while TDs that arose in Cape Verde increased in frequency in the population due to positive selection as a result of their role in enhancing B transport into leaf tissues. We previously observed a similar pattern at the *NRAMP1* locus in Fogo, where multiple independent TDs arose and collectively rose to high frequency across the structured population (45). In both cases, it is especially striking that so many independent TDs arose in the time since colonization of the islands, approximately 5 kya (27). Increases in gene copy number have been implicated in adaptive functions in metal hyperaccumulator plants (49) and more broadly in diverse plant, microbial, and animal species (50–52), including during domestication and local adaptation of crops (53). Taken together, our findings support the role of TDs in rapid trait evolution in natural populations.

This study leaves room for deeper investigation of the function of natural BOR variants and the ecological relevance of our findings. First, the nonsynonymous SNP we identified affects the core domain of *BOR2*, a gene that encodes a borate efflux transporter, but for which the role in B transport has been less clear. Based on homology to *BOR1*, where more molecular work has been done (18), we inferred that this SNP may inhibit the degradation of the BOR2 protein. Our findings are consistent with a previous study that showed that *bor2* mutants exhibited subtly decreased leaf B accumulation (30). Our results suggest that further investigation into the role of *BOR2* in B transport to leaf tissue and its regulation at the protein level would be worthwhile. Furthermore, our results imply that evolution has not increased passive B transport to leaf tissues in Cape Verdean plants but rather has acted on active transporters capable of transporting B against their concentration gradients. Second, we conducted our experiments using standard potting soil, but future experiments in native Cape Verde soils would provide the possibility to estimate heritabilities and variant effect sizes under more ecologically relevant conditions (54, 55).

In conclusion, this study provides insights into the genetic mechanisms underlying B accumulation and their evolution in natural populations and reveals a case of rapid convergent evolution. Specifically, SNPs and TDs at *BOR* efflux transporter genes played a critical role in driving increased leaf B transport in *A. thaliana* populations native to the drought-prone volcanic islands of Cape Verde. Furthermore, our finding that multiple independent TDs at *BOR1* arose in the last few thousand years population further highlights the role of TDs in promoting rapid evolutionary change and the importance of considering such variants in mapping studies.

## Materials and Methods

### Plant Material.

For mapping, we used 126 accessions from Fogo, 123 accessions from Santo Antão (27), and 167 individuals from the CVI intercross population developed in (35).

### Leaf B analysis.

To characterize leaf B content in the CVI natural and intercross populations, we applied a method similar to that used in (45). The seeds were stratified for 7 d at 4 °C in the dark on petri dishes supplemented with 800 µl of GA4/7 (100 µM). We then sowed the seeds on 54-well trays (60 cm by 40 cm by 6 cm) filled with standard potting mix (Einheits erde Special Type Min Tray substrate) and fertilized with Osmocote start 11-11-7 + 2MgO + TE fertilizer (1 g L^−1^). We propagated four replicates for each accession in a randomized block design. The seedlings were grown under controlled growth chamber conditions with 8 h of light, 21 °C (day), 14 °C (night). Five to six weeks after sowing, we harvested two to three young leaves per plant for ionomic analyses. To avoid soil contamination, we washed the leaves three times with 18.2 MÎ©.cm milli-Q water (Merck Millipore) before transferring them to 1.5 mL Eppendorf tubes. We then dried the leaf samples overnight at 80ºC. Leaf elemental content analysis was conducted using Inductively Coupled Plasma Mass Spectrometry (ICP-MS) as described in (56). In addition to B, we estimated Cr and Ti leaf contents to control for soil contamination. To estimate leaf B values, we first computed a z-score filtering on B, Cr, and Ti to remove individuals showing an absolute z-score above three, and removed accessions with less than three replicates after z-score filtering. Next, we accounted for block effects with the Best Linear Unbiased Estimates (BLUEs) using the lme4 package (version 1.1-35.5) (57) in R (version 4.4.2) (58).

### GWAS.

VCFs for the CVI natural and intercross populations (27, 35) were generated using GATK (59, 60). We filtered the VCFs for each population separately. For the natural populations, we used VCFtools (61) to include only biallelic variants where samples have a read depth (DP) greater than three and genotype quality (GQ) above 20. For the CVI-intercross population, the VCF was already filtered in (35).

We then retained variants with a minor allele frequency below 5% and missingness below 10%. To account for population structure, we generated a centered kinship matrix using the -gk 1 parameter in GEMMA. We characterized the genetic architecture underlying leaf B variation as in (62). We estimated the broad-sense heritability (H^2^) with the following equation:

$$H^{2}=\frac{{\mathrm{variance}}_{\mathrm{line}}}{{\mathrm{variance}}_{\mathrm{line}}+{\mathrm{variance}}_{\mathrm{residual}}},$$

using the lme4 package (version 1.1-35.5) (57). To estimate the narrow-sense heritability, we calculated the chip heritability using a Bayesian Sparse Linear Mixed Model (BSLMM) in GEMMA (version 0.94) (28). For this, we calculated the median and 95% CI for the proportion of variance in phenotypes explained by available genotypes (PVE) after running Markov chain Monte Carlo (MCMC) with 10 000 000 sampling steps and 2 500 000 burn-in iterations across ten runs. We examined the potential candidate variants based on the results of the LMM we ran in GEMMA (version 0.94) (29). To estimate the effect size of a variant, we used beta, which represents the unit change of the phenotype per copy of the minor allele. To enable comparison between homozygous individuals, we reported twice the beta value. To assess excess of significant p-values, we conducted genomic control with SciPy (version 1.6.2) using the following equation:

$$\lambda =\frac{O\left(\chi_{\mathrm{median}}^{2}\right)}{E\left(\chi_{\mathrm{median}}^{2}\right)},$$

where λ corresponds to the genomic control, $O\left(\chi_{\mathrm{median}}^{2}\right)$ is the observed median of the chi-squared values, and $E\left(\chi_{\mathrm{median}}^{2}\right)$ is 0.4549, the expected median of a chi-squared distribution with one degree of freedom. We estimated pairwise linkage disequilibrium using PLINK (version v1.90b6.26) (63) with the following parameters: --ld-window-kb 1000, --ld-window 99999, --ld-window-r2 0.

### Protein Structure Predictions.

The structural predictions of BOR1 and BOR2 were generated in AlphaFold2 (31) and visualized with ChimeraX (version 1.9) (64). We used Missense3D to evaluate the effect of the *BOR2 313G* mutation on the predicted structure of BOR2 (65).

### Multiple Alignment.

The multiple alignment was generated with the muscle algorithm (66) and visualized with Jalview (67). We used the following sequences (Uniprot IDs): AtBOR1 (Q8VYR7), AtBOR2 (Q9M1P7), AtBOR3 (Q93Z13), AtBOR4 (Q9XI23), AtBOR5 (Q9SSG5), AtBOR6 (Q3E954), AtBOR7 (Q9SUU1), BnBOR1 (D5LG95), BnBOR2 (D5LG96), ZmRTE (B6U4C0), ZmBOR2_1 (A0A8J8YEE5), ZmBOR2_2 (A0A3L6FE15), and HaBOR1 (HannXRQ_Chr10g0285571).

### *BOR1* Copy Number Estimation.

To estimate the number of *BOR1* copies in F13-8 based on long-read sequencing data, we used the F13-8 de novo assembly generated in (45). Genomic DNA was extracted from pooled leaf tissue, and DNA fragments larger than 30 kb were selected using BluePippin (Sage Science). Libraries were prepared with the Ligation Sequencing Kit 1D (Oxford Nanopore Technologies, catalog No SQK-LSK109) and sequenced on a GridION X5 platform (Oxford Nanopore Technologies). The F13-8 de novo assembly was generated using miniasm-0.3 (68) and minimap2-2.17. The resulting draft assembly was polished twice with racon (version 1.4.10) (69) using the raw Nanopore reads, followed by ten rounds of polishing with pilon (version 1.23) (70) using Illumina short reads. To orient and scaffold the contigs, we used the reference genome assembly (TAIR10). To prevent Illumina short reads from mapping to the anchoring points of the contigs, we inserted 1,000 “N”s between the contigs. The complete pipeline can be found at https://github.com/HancockLab/Fogo-Edaphic. We then aligned the F13-8 de novo assembly to the reference genome assembly (TAIR10) with the nucmer package from MUMmer (version 3.1) (71) using the following parameters: --maxmatch, -l 100, -c 500. To identify structural variants, we used Assemblytics (version 1.2) (72) with the following parameters: a unique sequence length required of 1,000 bp, a maximum variant size of 1,00,000 bp, and a minimum variant size of 50 bp.

To estimate *BOR1* CN based on short-read sequenced genomes, we applied the same method as in (45). We used Delly (version 0.8.3) (73) to identify the breakpoint positions at the tandem duplications. We then used the “--depth” command in VCFtools (version 0.1.16) (61) to obtain the coverage at each base pair between the positions 19328767 and 19392651 on chromosome 2. We calculated the sequencing depth in 800 bp sliding windows every 400 bp and normalized it to the 3 kb regions upstream and downstream of the breakpoints with a Python (version 3.8.3) custom script, and reported the median as an estimate for *BOR1* CN.

To assess *BOR1* CN by digital PCR (dPCR), we extracted DNA from leaf tissue using the DNeasy Plant Mini Kit (Qiagen, catalog no. 69106). The genomic DNA was quantified using the Qubit fluorometer (Thermo Fisher Scientific, catalog no. Q33238). We conducted the dPCR with the QIAcuity five-plex device (Qiagen, catalog no. 911001) using QIAcuity Nanoplates 26 K 24-well plates (Qiagen, catalog no. 250021). We set the dPCR mixtures in duplex using the QIAcuity Probe PCR Kit (Qiagen, catalog No. 250101) according to the manufacturer’s instructions, with 5 ng of genomic DNA and 0.2 unit of EcoRI-HF (NEB). The thermal cycling conditions consisted of an initial heat activation of 2 min at 95 °C, followed by 40 cycles of denaturation for 15 s at 95 °C and annealing/extension for 15 s at 55 °C. *PHOSPHATASE 2A (PP2A)* (AT1G13320) was used as a reference gene after excluding the presence of extra copies with IGV (74). We used the probe P1 (5’ FAM, BHQ1 3’) with primers P2 and P3 for *BOR1* and the probe P4 (5’ ROX, BHQ2 3’) with primers P5 and P6 for *PP2A*. Primers and probes described here are detailed in Dataset S10. FAM was detected on the green channel with an exposure time of 500 ms and a gain of 6. ROX was detected on the red channel with an exposure time of 300 ms and a gain of 4.

### *BOR1* Expression Analysis.

We assessed *BOR1* expression in root tissues as described in (45). The seeds were sterilized with 70% ethanol and stratified for 7 d at 4 °C in the dark on square 120-mm polystyrene petri dishes (Greiner) filled with (1×) Murashige and Skoog (MS). The plates were then positioned vertically in a growth chamber (Percival Scientific) under long-day conditions (16 h of light, 8 h of dark, 21 °C). Root tissues from 15 seedlings were collected 14 d after sowing. We conducted RNA extraction using TRIzol (Invitrogen, catalog no. 15596026). To prevent any DNA contamination, we treated one microgram of RNA with the DNA-free DNA Removal Kit (Invitrogen, catalog no. AM1906) for 1 h at 37 °C, according to the manufacturer’s instructions. To generate complementary DNA, we used the SuperScript IV reverse transcriptase (Invitrogen, catalog no. 18090050) with an oligo(dT)18 following the manufacturer’s protocol. We quantified mRNA levels by dPCR as described in (45). We used the QIAcuity five-plex device (Qiagen, catalog no. 911001) with QIAcuity Nanoplates 26 K 24-well plates (Qiagen, catalog no. 250021). We set the dPCR mixtures in duplex with the QIAcuity Probe PCR Kit (Qiagen, catalog no. 250101) according to the manufacturer’s instructions. For normalization, we used *PP2A* as a reference gene and F3-2 as a control genotype. We used the probe P7 (5′ FAM, BHQ1 3′) with primers P8 and P9 for *BOR1* and the probe P4 (5′ ROX, BHQ2 3′) with primers P5 and P6 for *PP2A*. Primers and probes used in this study are detailed in Dataset S10. Thermocycling conditions and fluorophore detection settings were the same as for *BOR1* copy number estimation.

### Reconstruction of the Evolutionary Histories of *BOR1* and *BOR2* in Cape Verde.

To infer genealogical trees at *BOR1* and *BOR2*, we used Relate (version 1.1.4) (44) as described in (45). In Fogo, we used as an outgroup S1-1 and S8-502, two accessions originating from subpopulations in Santo Antão that are genetically the closest to the Fogo population (27). We filter the VCF to only include biallelic SNPs where all samples have DP above 3 and GQ above 25. For the TDs at *BOR1*, we introduced variants into the hap file corresponding to the breakpoints, as shown in (Fig. 5*A*). We ran Relate under a haploid model. Since Relate does not allow missing data, we corrected the mutation rate to account for missingness across the chromosome. Specifically, we scaled the spontaneous mutation rate previously estimated in *A. thaliana* (75) with the following equation:

$$7\times 10^{-9}\left(1-\frac{missing\;variants}{total\;variants}\right),$$

where total variants correspond to biallelic SNPs with at most 10 % missingness, and all remaining samples have DP above 3 and GQ above 25. We conducted this analysis using 10 Mb sliding windows with 50 kb step sizes. In Santo Antão, the corrected mutation rate was 1.91192 × 10^−9^ for *BOR1* chr2:19374681 and 1.90843 × 10^−9^ for the *BOR1* TD (chr2: chr2:19320841). In Fogo, we obtained 2.82893 × 10^−9^ for *BOR2* and 2.77543 × 10^−9^ for the *BOR1* TD haplotypes. To correct for the outcrossing rate of 5% estimated in natural populations (76), we used a previously described recombination map (77), where we divided the genetic distances by twenty. We set the generation time to one year. We calculated age and CI estimates across 200 MCMC runs using the “SampleBranchLength.sh” script.

### Geographical Maps.

Every map was generated using R (version 3.6.2). We used Rayshader (version 0.38.1) to represent the natural population (78). For the geographical distribution of the *BOR1* and *BOR2* haplotypes, we used Stadia Maps (https://stadiamaps.com/), OpenMapTiles (https://openmaptiles.org/), OpenStreetMap (https://www.openstreetmap.org/copyright), and Stamen Design (https://stamen.com/).

## Supplementary Material

Appendix 01 (PDF)

Dataset S01 (TXT)

Dataset S02 (TXT)

Dataset S03 (TXT)

Dataset S04 (TXT)

Dataset S05 (TXT)

Dataset S06 (TXT)

Dataset S07 (TXT)

Dataset S08 (TXT)

Dataset S09 (TXT)

Dataset S10 (TXT)

Dataset S11 (XLSX)

Dataset S12 (XLSX)

## Data Availability

Nanopore data generated for this study have been deposited in NCBI SRA under the project number PRJNA1112600 (79). Source data for each figure can be found in Datasets S11 and S12. All scripts for analyses and data visualization can be found in the GitHub repository (https://github.com/HancockLab/CVI-Boron) (80).
