# Supplementary material for: Convergent evolution increases boron transport through SNPs and tandem duplications at BOR1 and BOR2 in Arabidopsis thaliana
Source: Proc Natl Acad Sci U S A. 2026 Mar 23;123(13):e2525676123. doi: 10.1073/pnas.2525676123 (PMC13037888; doi:10.1073/pnas.2525676123)
Supplement: Supplementary file 1 — Appendix 01 (PDF) [file pnas.2525676123.sapp.pdf]

PNAS

**Supporting Information for**

# Convergent evolution increases boron transport through SNPs and tandem duplications at *BOR1* and *BOR2* in *Arabidopsis thaliana*

Emmanuel Tergemina, Célia Neto, Md Mamunur Rashid, Herculano Dinis, David E. Salt and Angela M. Hancock

Angela M. Hancock

Email: ahancock@purdue.edu

**This PDF file includes:**

Figures S1 to S15

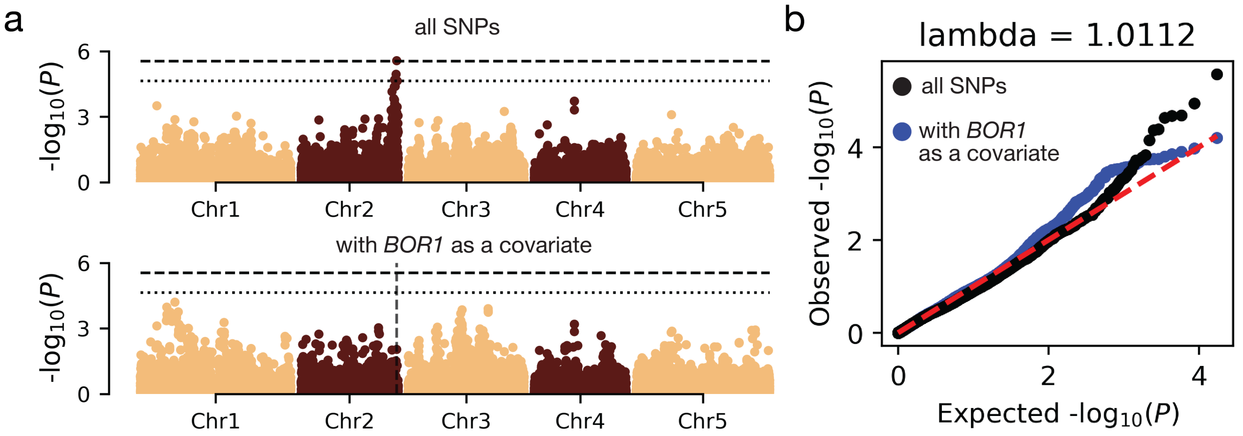


**Supplementary Figure 1: GWAS for variation in leaf B in Santo Antão reveals a peak at *BOR1.***

**a**, The upper panel corresponds to the unconditional GWAS. The lower panel corresponds to the GWAS after conditioning for the *BOR1* region (chr2:19374681). The dashed horizontal line corresponds to the 5% Bonferroni-adjusted genome-wide significance threshold. The dotted horizontal lines indicate the 10% false discovery rate (FDR) threshold. The vertical dashed line indicates the marker added in the LMM for the conditional analysis (chr2:19374681). **b**, Quantile-quantile plot showing the relationship between observed and expected associations with leaf B content in Santo Antão. The red line indicates the expected relationship under the null hypothesis.

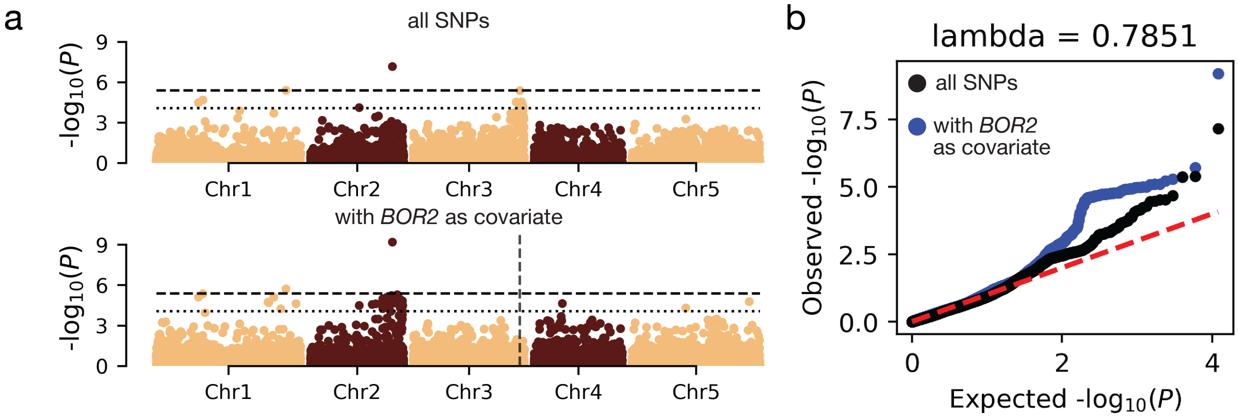


**Supplementary Figure 2: GWAS for variation in leaf B in Fogo reveals a peak at *BOR2*.**

**a**, The upper panel corresponds to the unconditional GWAS. The lower panel corresponds to the GWAS after conditioning for the *BOR2* region (chr3:22436584). The dashed horizontal line corresponds to the 5% Bonferroni-adjusted genome-wide significance threshold. The dotted horizontal lines indicate the 10% false discovery rate (FDR) threshold. The vertical dashed line indicates the marker added in the LMM for the conditional analysis (chr3:22436584). **b**, Quantile-quantile plot showing the relationship between observed and expected associations with leaf B content in Fogo. The red line indicates the expected relationship under the null hypothesis.

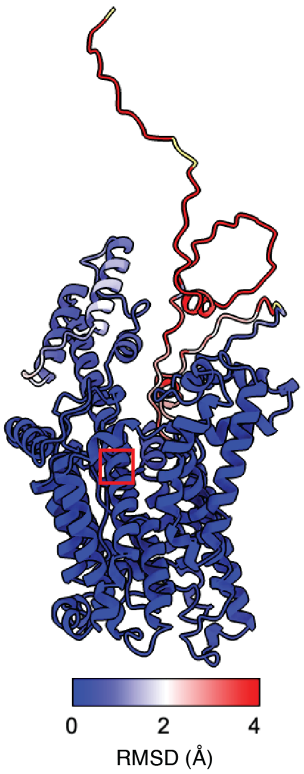


**Supplementary Figure 3: BOR1 and BOR2 have a similar structure.**

Predicted structure of BOR2 with the root mean square deviation of the C alpha atomic coordinates from the predicted structure of BOR1. BOR2 S313 is indicated with a red rectangle.

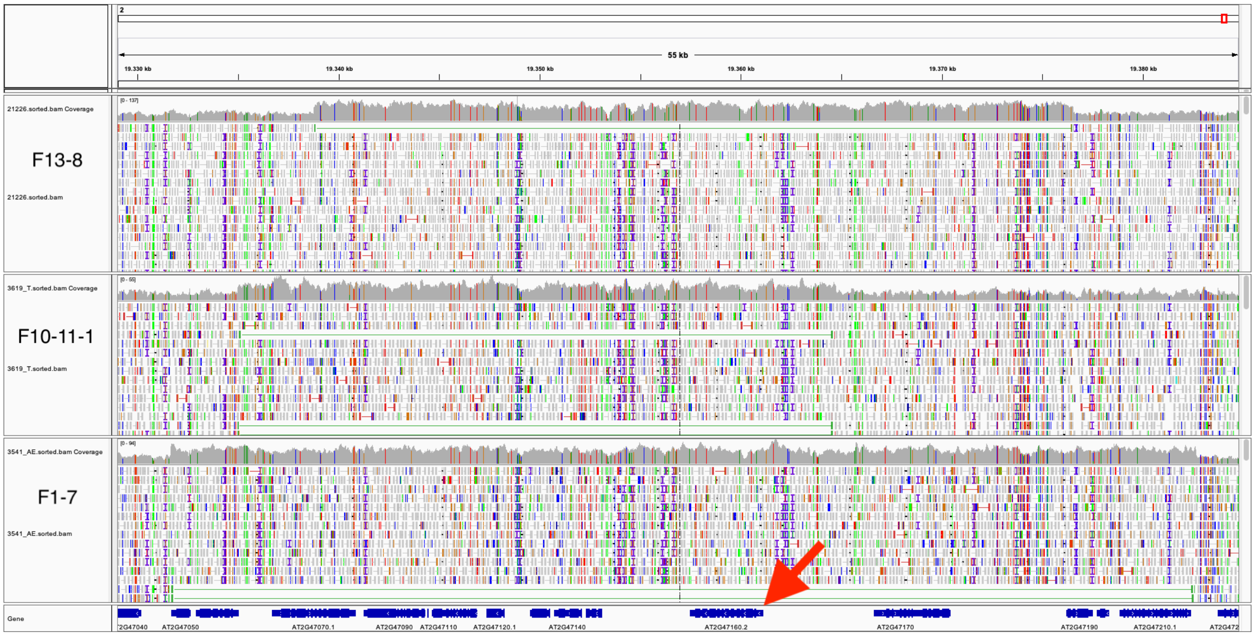


**Supplementary Figure 4: *BOR1* TD haplotypes in the Fogo population.**

IGV browser view at the *BOR1* genomic region showing the TD in F13-8 (chr2:19338767 haplotype), F10-11-1 (chr2:19334984 haplotype), and F1-7 (chr2:19331669 haplotype). *BOR1* is indicated with a red arrow. The reads are shown as pairs and aligned to the TAIR10 reference genome.

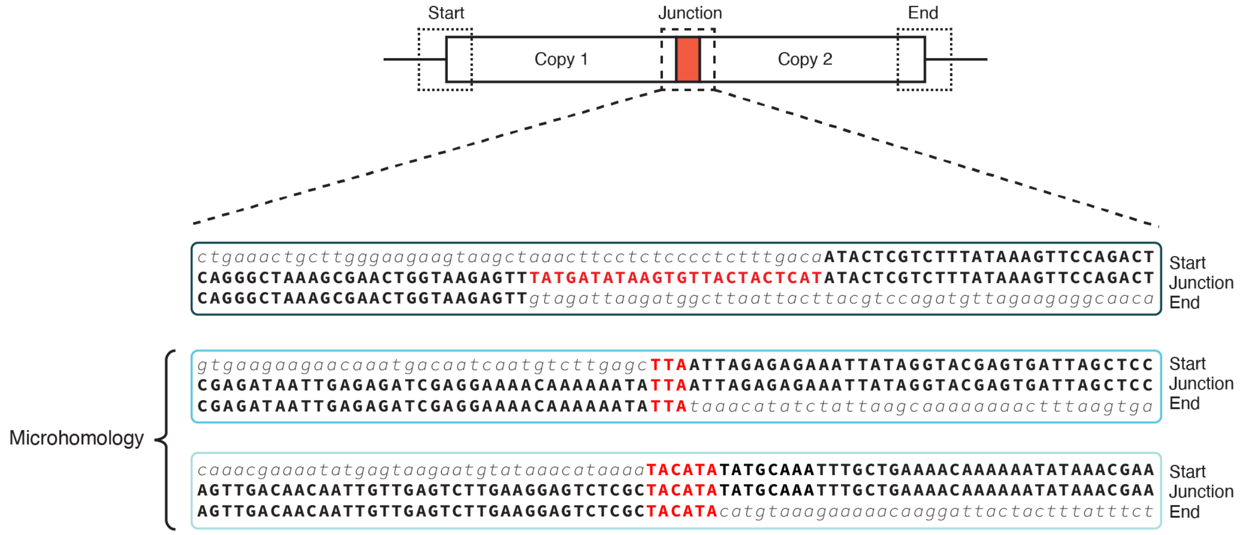


**Supplementary Figure 5: Breakpoint junctions at the TDs surrounding *BOR1*.**

Breakpoint junctions (in red) at the TD surrounding *BOR1*. One *BOR1* TD haplotype (chr2:19338767, Supplementary Figure 6) contains a 26 bp junction (*TATGATATAAGTGTTACTACTCAT*). Two *BOR1* TD haplotypes, chr2:19334984 (Supplementary Figure 7) and chr2:19331669 (Supplementary Figure 8), show two different microhomology-mediated junctions (*TTA* and *TACATA*). The breakpoint junctions are aligned to the start and the end of the breakpoints.

a

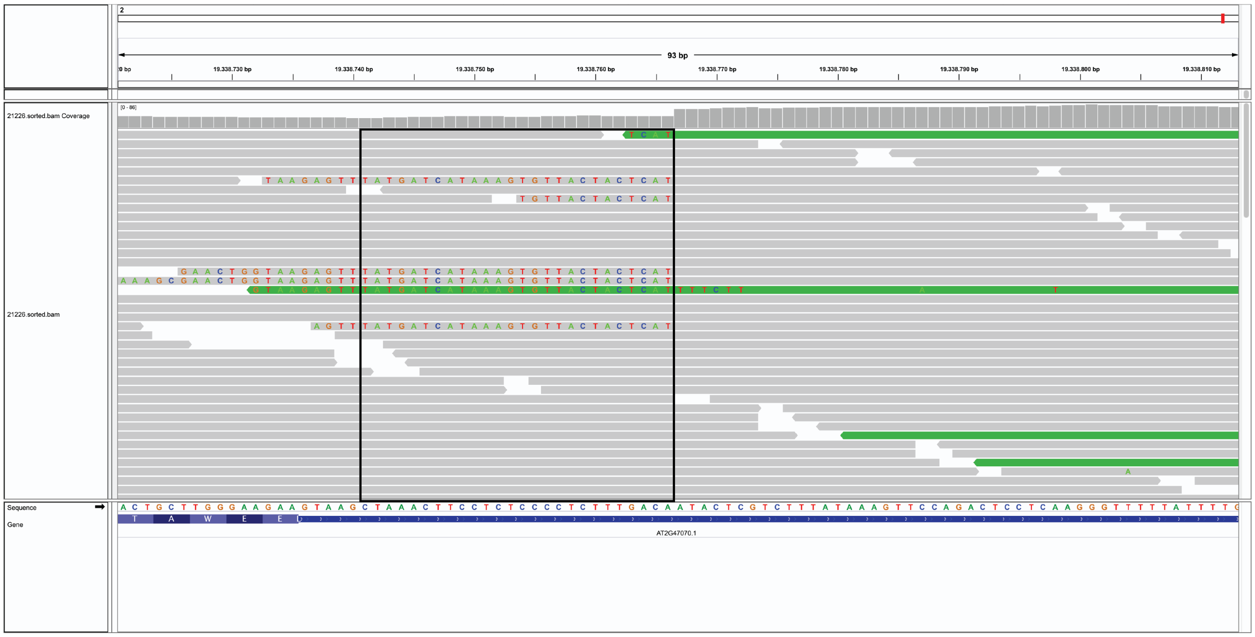


b

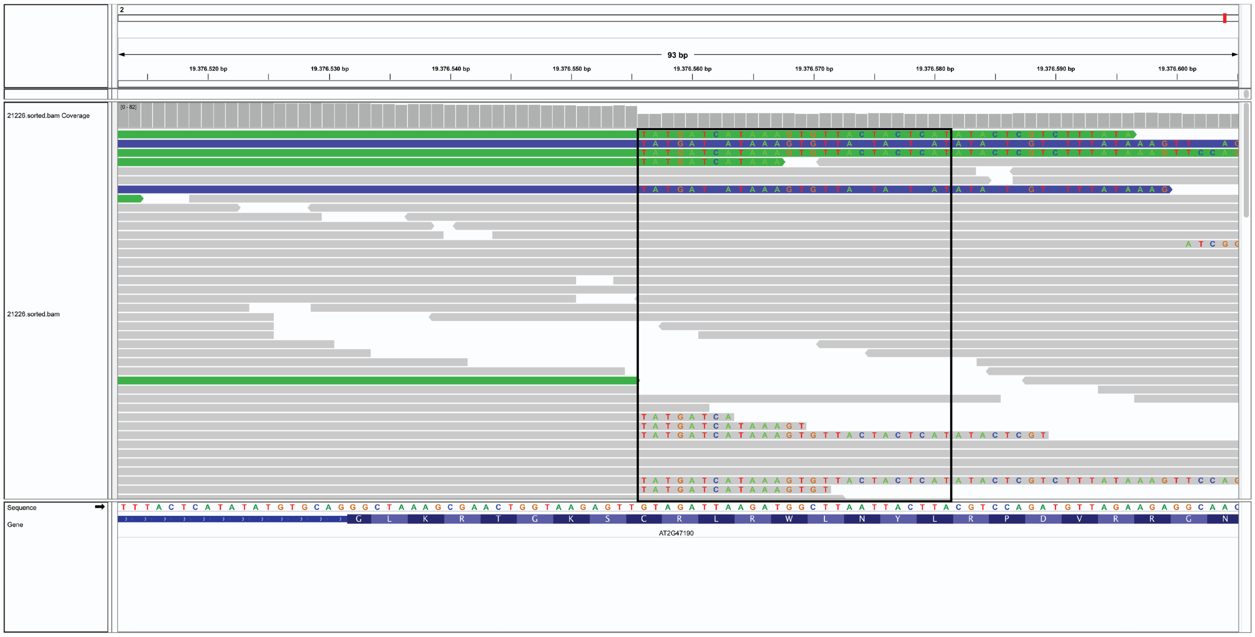


**Supplementary Figure 6: Breakpoints at the *chr2:19338767* TD haplotype in F13-8.**

IGV browser view of the breakpoints upstream (a) and downstream (b) of the tandem duplication surrounding *BOR1* showing the discordant reads (in green) and the soft-clipped bases at the breakpoints (multicolor). The black rectangles indicate the inserted sequence at the TD junction. The blue reads in (b) correspond to a read-pair mapping directly to the junction at the TD. They appear highlighted in blue in IGV because, as a

result of the insertion at the TD junction, they are mapped closer together than expected on the reference genome.

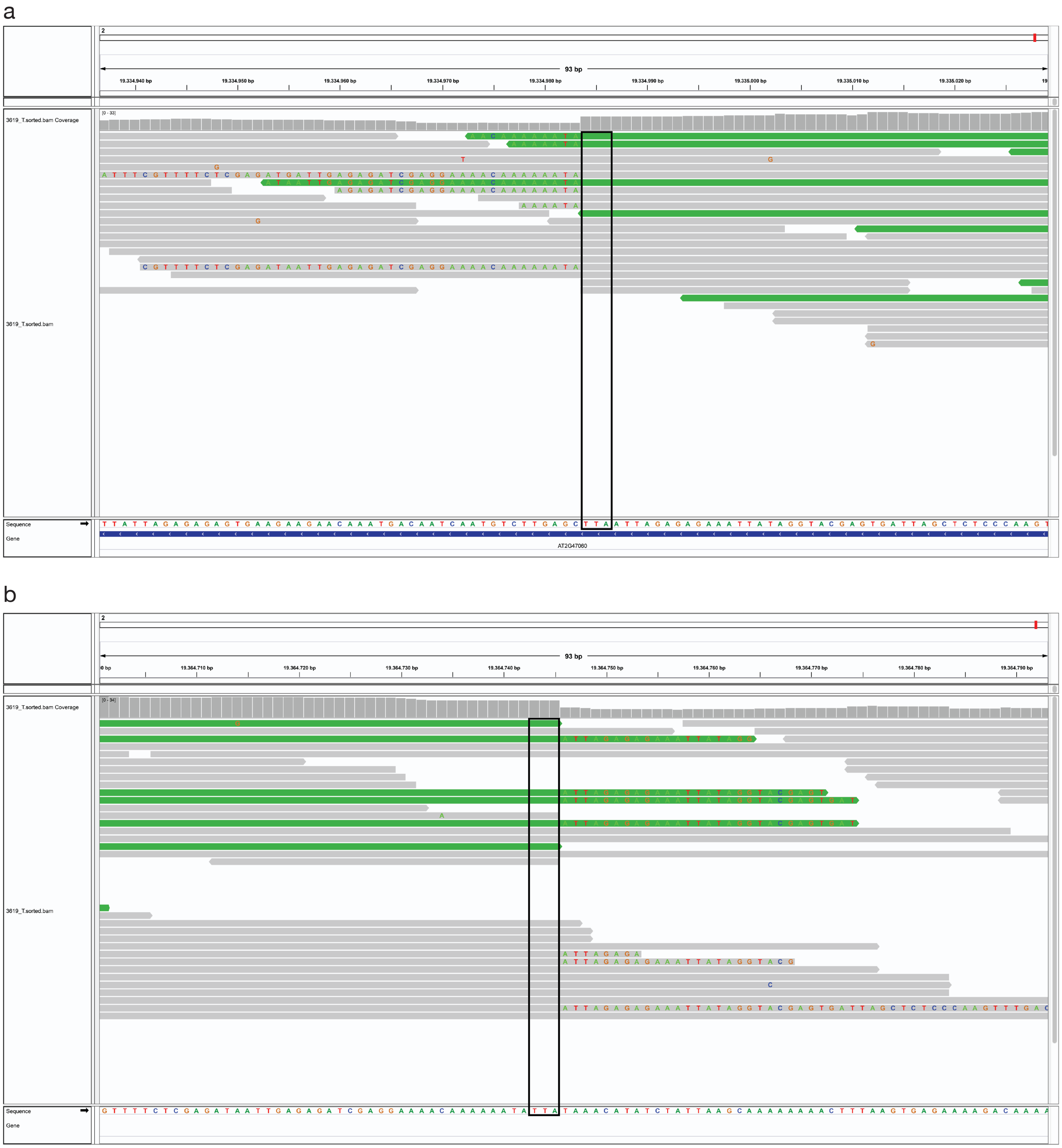


**Supplementary Figure 7: Breakpoints at the *chr2:19334984* TD haplotype in F10-11-1.**

IGV browser view of the breakpoints upstream (a) and downstream (b) of the tandem duplication surrounding *BOR1* showing the discordant reads (in green) and the soft-clipped bases at the breakpoints (multicolor). The black rectangles indicate the microhomology at the TD junction.

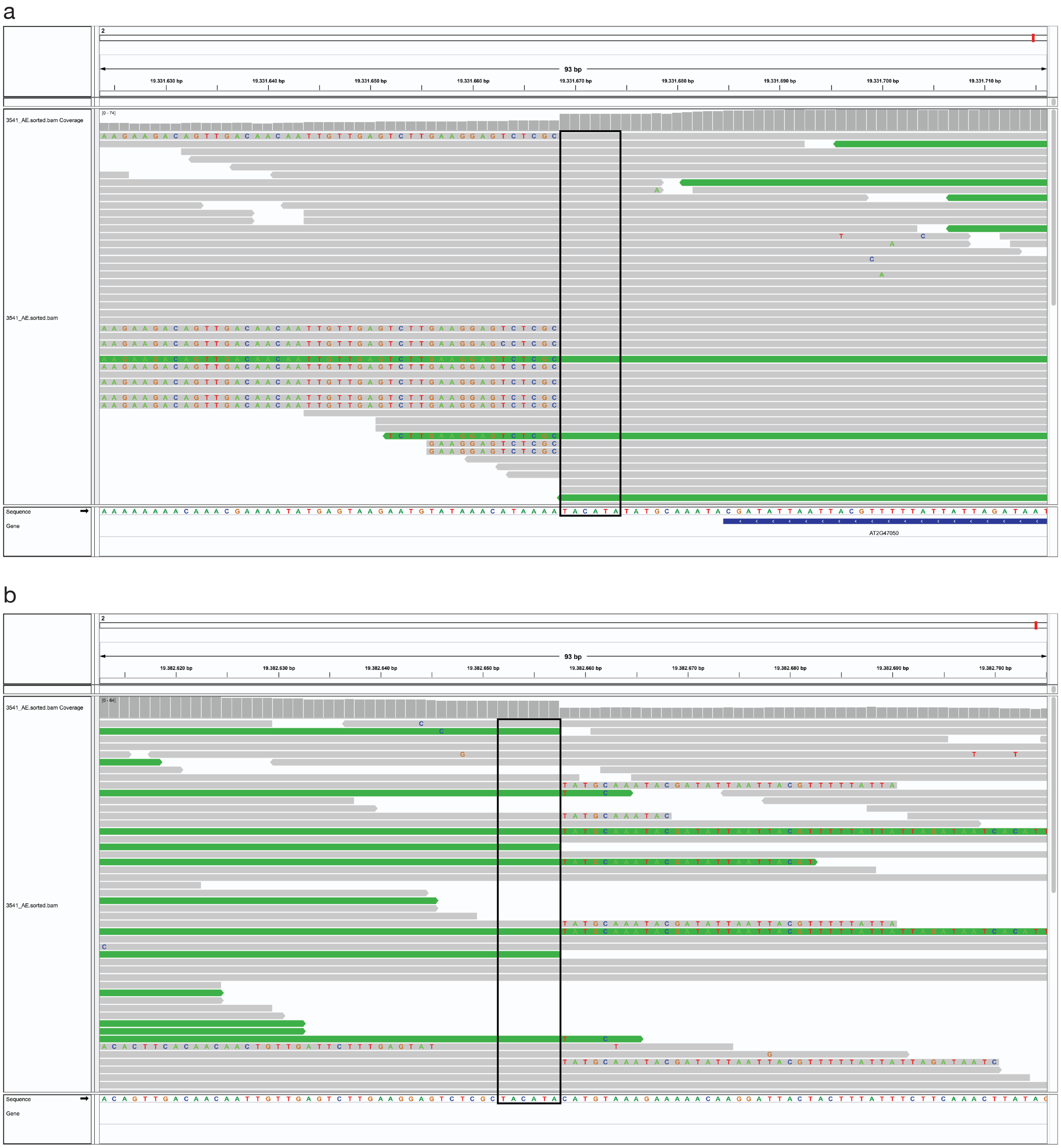


**Supplementary Figure 8: Breakpoints at the *chr2:19331669* TD haplotype in F1-7.**

IGV browser view of the breakpoints upstream (a) and downstream (b) of the tandem duplication surrounding *BOR1* showing the discordant reads (in green) and the soft-clipped bases at the breakpoints (multicolor). The black rectangles indicate the microhomology at the TD junction.

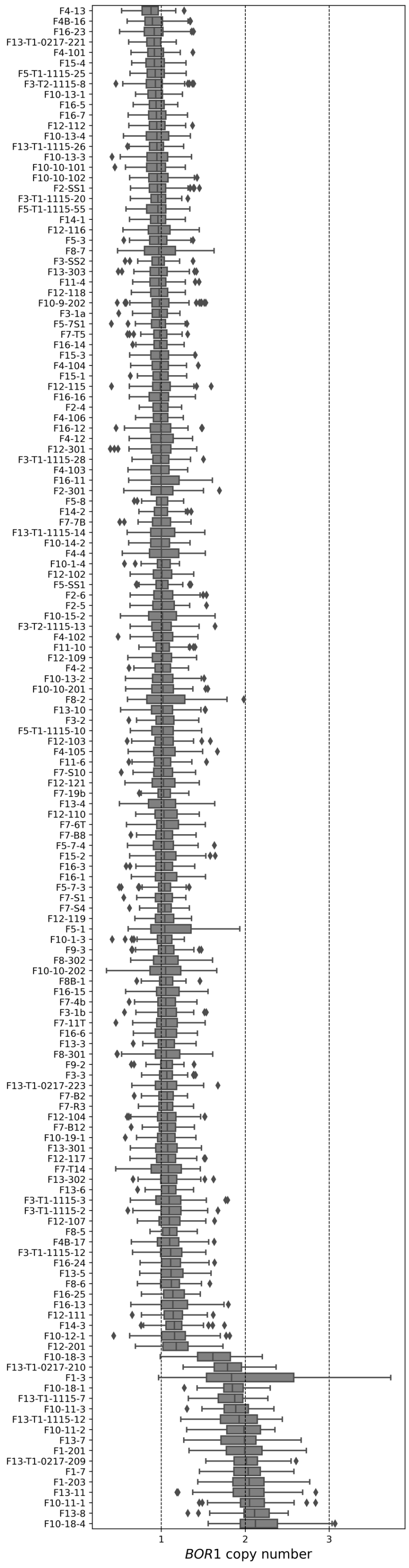
F4-13
F4B-16
F16-23
F13-T1-0217-221
F4-101
F15-4
F5-T1-1115-25
F3-T2-1115-8
F10-13-1
F16-5
F16-7
F12-112
F10-13-4
F13-T1-1115-26
F10-13-3
F10-10-101
F10-10-102
F2-SS1
F3-T1-1115-20
F5-T1-1115-55
F14-1
F12-116
F5-3
F8-7
F3-SS2
F13-303
F11-4
F12-118
F10-9-202
F3-1a
F5-7S1
F7-T5
F16-14
F15-3
F4-104
F15-1
F12-115
F16-16
F2-4
F4-106
F16-12
F4-12
F12-301
F3-T1-1115-28
F4-103
F16-11
F2-301
F5-8
F14-2
F7-7B
F13-T1-1115-14
F10-14-2
F4-4
F10-1-4
F12-102
F5-SS1
F2-6
F2-5
F10-15-2
F3-T2-1115-13
F4-102
F11-10
F12-109
F4-2
F10-13-2
F10-10-201
F8-2
F13-10
F3-2
F5-T1-1115-10
F12-103
F4-105
F11-6
F7-S10
F12-121
F7-19b
F13-4
F12-110
F7-6T
F7-B8
F5-7-4
F15-2
F16-3
F16-1
F5-7-3
F7-S1
F7-S4
F12-119
F5-1
F10-1-3
F9-3
F8-302
F10-10-202
F8B-1
F16-15
F7-4b
F3-1b
F7-11T
F16-6
F13-3
F8-301
F9-2
F3-3
F13-T1-0217-223
F7-B2
F7-R3
F12-104
F7-B12
F10-19-1
F13-301
F12-117
F7-T14
F13-302
F13-6
F3-T1-1115-3
F3-T1-1115-2
F12-107
F8-5
F4B-17
F3-T1-1115-12
F16-24
F13-5
F8-6
F16-25
F16-13
F12-111
F14-3
F10-12-1
F12-201
F10-18-3
F13-T1-0217-210
F1-3
F10-18-1
F13-T1-1115-7
F10-11-3
F13-T1-1115-12
F10-11-2
F13-7
F1-201
F13-T1-0217-209
F1-7
F1-203
F13-11
F10-11-1
F13-8
F10-18-4
1
2
3
*BOR*1 copy number

**Supplementary Figure 9: *BOR1* copy number variation in the Fogo population.**

*BOR1* copy number estimation from short-read sequencing data. *BOR1* copy number corresponds to the coverage at the breakpoints over the coverage at the 3-kb surrounding regions. We used 800-bp sliding windows with a step size of 400.

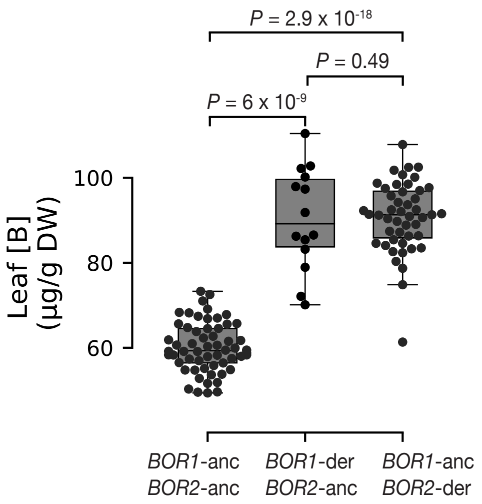


**Supplementary Figure 10: Variation in leaf B content in Fogo with allelic combination between *BOR1* and *BOR2*.**

Results are shown in µg/g of DW. Each dot represents an accession. anc = ancestral, der = derived. *P* = *P* value for MWW test. We did not observe any Fogo accession carrying *BOR1*-der and *BOR2*-der.

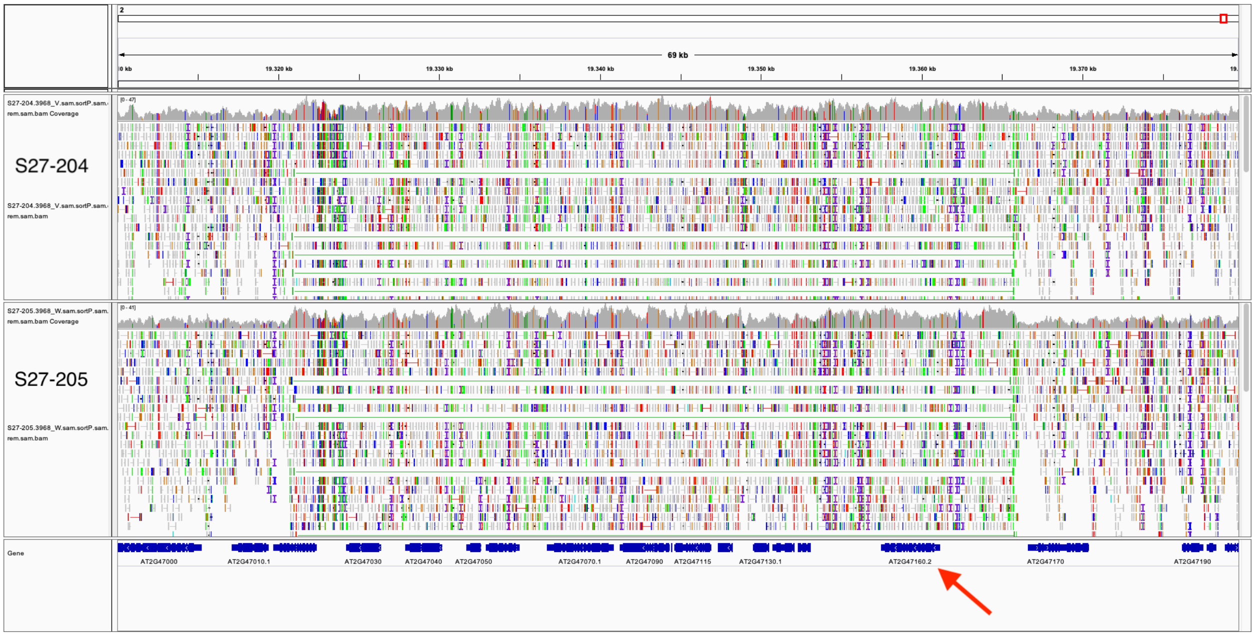


**Supplementary Figure 11: *BOR1* TD in Santo Antão.**

IGV browser view at the *BOR1* genomic region in S27-204 and S27-205. *BOR1* is indicated with a red arrow. The reads are shown as pairs and aligned to the TAIR10 reference genome.

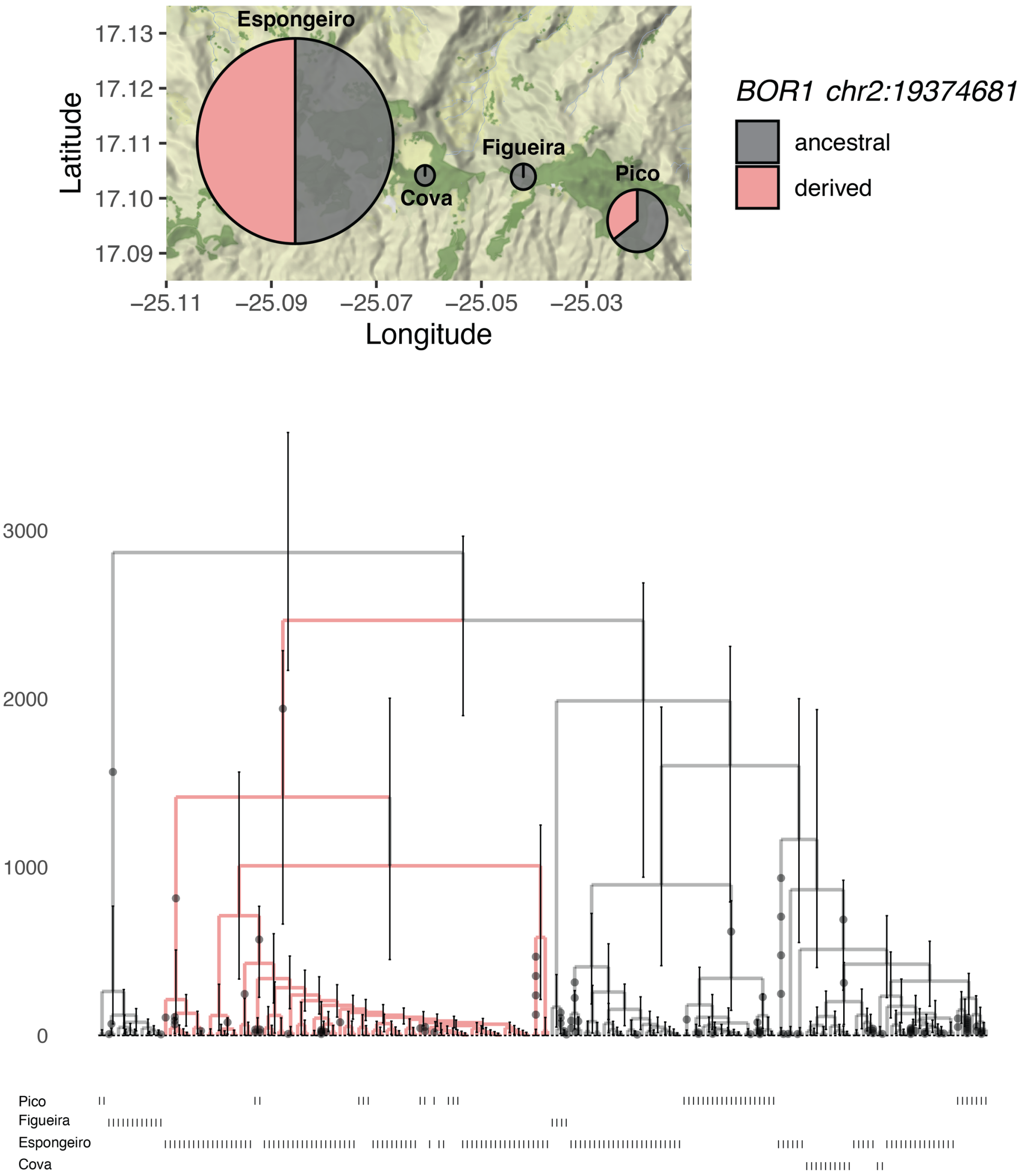


**Supplementary Figure 12: Evolutionary history of *BOR1 chr2:19374681* in Santo Antão.**

**a**, Geographical distribution of the *BOR1 chr2:19374681* variant in Santo Antão. **b**, Marginal tree at *BOR*1 in Santo Antão*.* Individuals are shown on the x-axis. Black ticks represent individuals with their corresponding populations indicated on the left side. The ancestral *BOR1* allele is colored in black and the derived *BOR1 chr2:19374681* allele is colored in red.

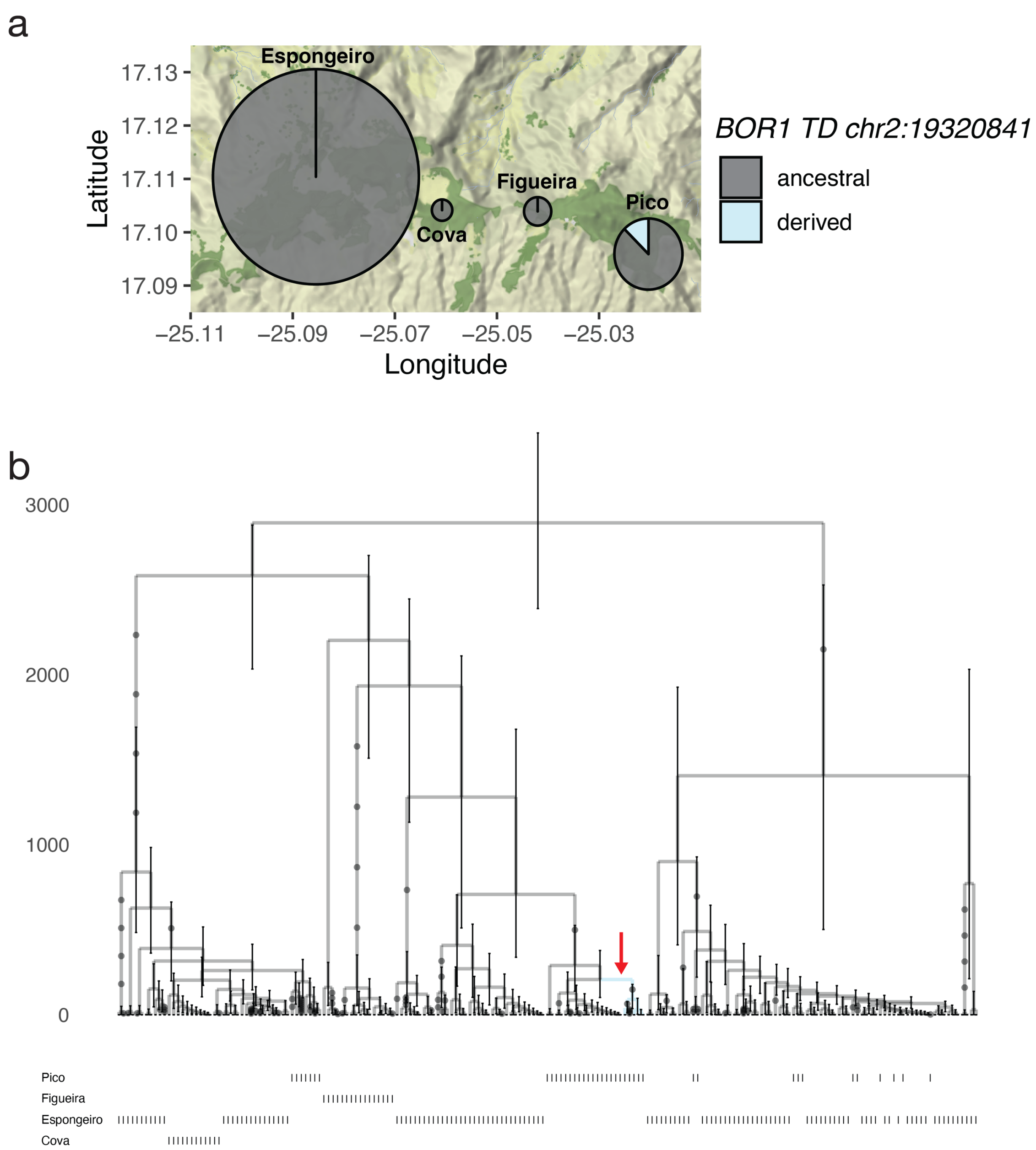


**Supplementary Figure 13: Evolutionary history of *BOR1 TD chr2:19320841* in Santo Antão.**

**a**, Geographical distribution of the *BOR1 TD chr2:19320841* variant in Santo Antão. **b**, Marginal tree at *BOR1* in Santo Antão. Individuals are shown on the x-axis. Black ticks represent individuals with their corresponding populations indicated on the left side. The ancestral *BOR1* allele is colored in gray and the derived *BOR1 TD chr2:19320841* allele is colored in light blue and indicated with a red arrow.

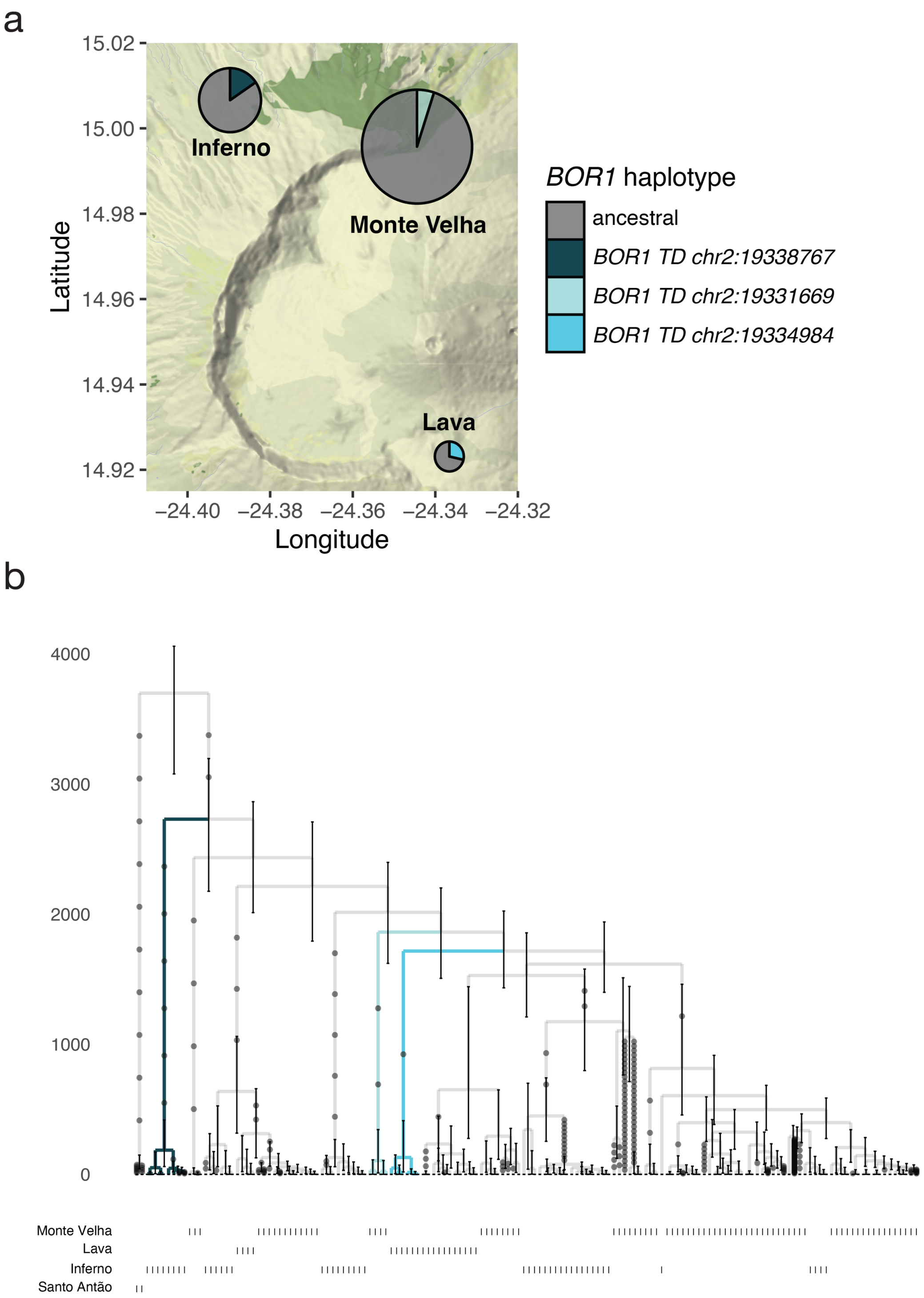


**Supplementary Figure 14: Evolutionary history of *BOR1* TDs in Fogo.**

**a**, Geographical distribution of the *BOR1* TDs in Fogo. **b**, Marginal tree at *BOR1* in Fogo. Individuals are shown on the x-axis. Black ticks represent individuals with their corresponding populations indicated on the left side. The ancestral *BOR1* allele is

colored in gray and the derived *BOR1* TD alleles are colored with different blue colors. We chose as outgroup two accessions from Santo Antão (S1-1 and S8-1).

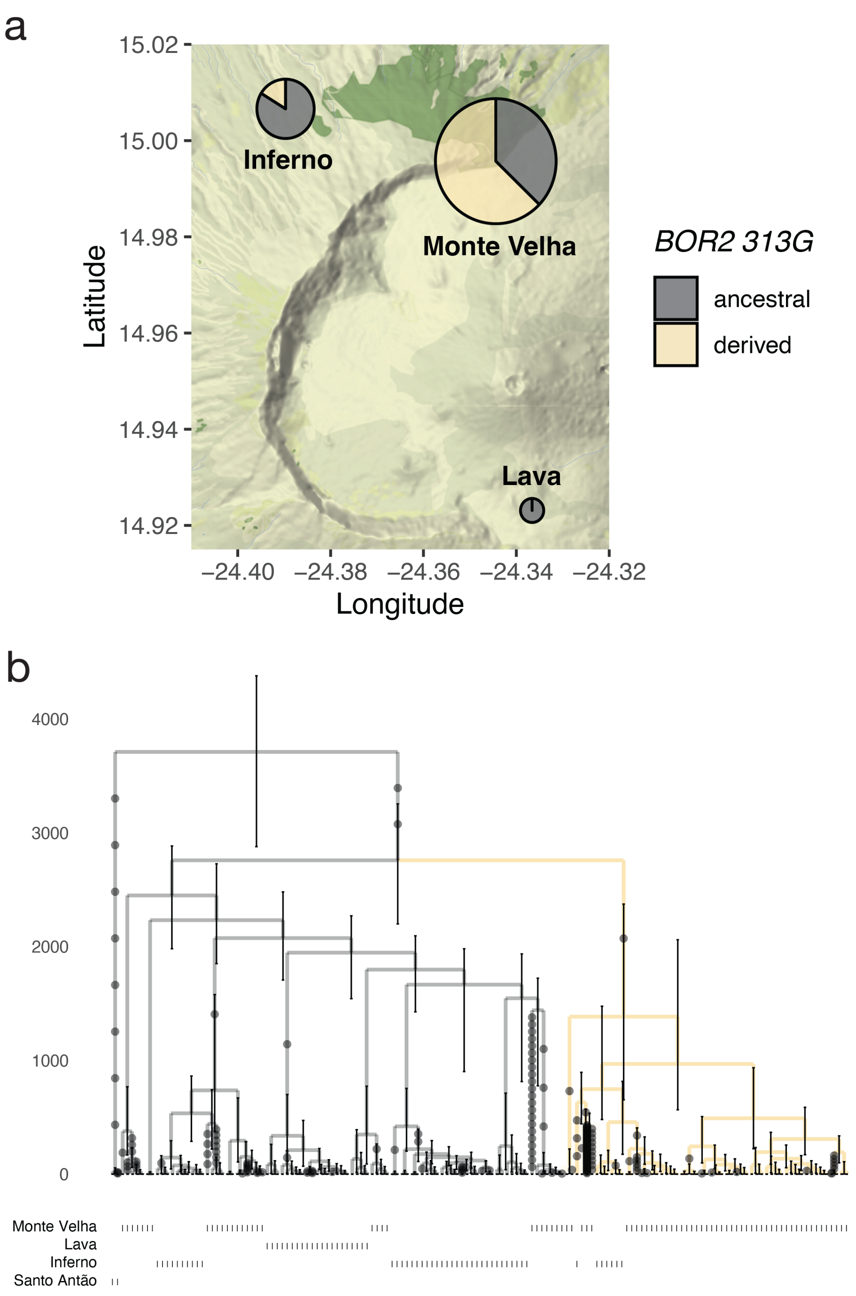


**Supplementary Figure 15: Evolutionary history of *BOR2 313G* in Fogo.**

**a**, Geographical distribution of the *BOR2 313G* variant in Fogo. **b**, Marginal tree at *BOR*2 in Fogo*.* Individuals are shown on the x-axis. Black ticks represent individuals with their corresponding populations indicated on the left side. The ancestral *BOR2* allele is colored in gray and the derived *BOR2 S313G* allele is colored in beige. We chose S1-1 and S8-1 as outgroup, two accessions from the closest relative (Santo Antão).
